# Supplementary material for: Structure of BRCA1-BRCT/Abraxas Complex Reveals Phosphorylation-Dependent BRCT Dimerization at DNA Damage Sites
Source: Mol Cell. 2016 Feb 4;61(3):434–48. doi: 10.1016/j.molcel.2015.12.017 (PMC4747905; doi:10.1016/j.molcel.2015.12.017)
Supplement: Document S1. Supplemental Experimental Procedures and Figures S1–S6 [file mmc1.pdf]

Molecular Cell

Supplemental Information

Structure of BRCA1-BRCT/Abraxas Complex Reveals Phosphorylation-Dependent BRCT Dimerization at DNA Damage Sites

Qian Wu, Atanu Paul, Dan Su, Shahid Mehmood, Tzeh Keong Foo, Takashi Ochi, Emma L. Bunting, Bing Xia, Carol V. Robinson, Bin Wang, and Tom L. Blundell

Supplemental Information

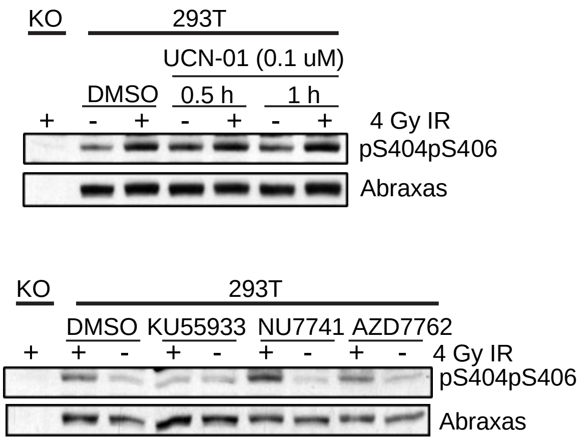

Figure S1 (related to Figure 1): IR-induced double phosphorylation of Abraxas C-terminus S404 and S406 is ATM-dependent, not ATR-, DNAPK-, Chk1- or Chk2 -dependent. Cells were incubated with Chk1 inhibitor (UCN-01), Chk1/2 inhibitor (AZD7762, 10 uM), ATM inhibitor (KU55933, 10 uM), DNA-PK inhibitor (NU7741, 10uM) for 2 hr at indicated concentrations. Cells were then either exposed to 4 Gy IR or untreated followed by 1 hr incubation at 37°C. Abraxas pS404pS406 levels were determined by Western blot.

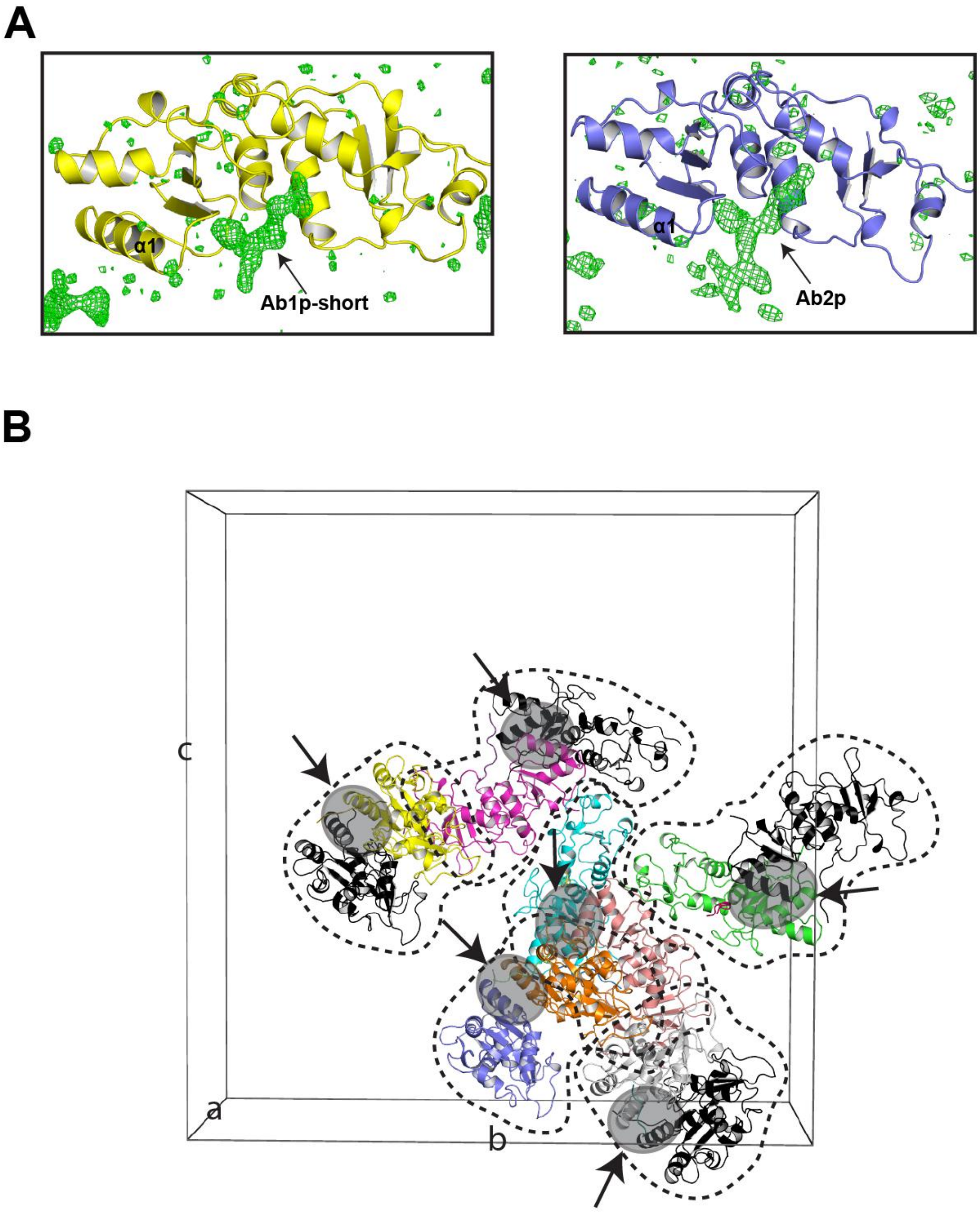

Figure S2 (related to Figures 2 and 3): Electron density for Abraxas phosphopeptide and arrangement of BRCT-Ab2p molecules in the crystal asymmetric unit. A) *Fo-Fc* map (σ=3.0) is shown for Ab1p_short and Ab2p. B) 8 copies of BRCT-Ab2p in one asymmetric unit of the cell (space group $P2_12_12_1$) were coloured in yellow, magenta, cyan, tint, green, orange, blue and grey individually. The same dimer interface occurs in each dimer of the BRCT-Ab2p complex. BRCT-Ab2p molecules belong to different ASUs are coloured in black. Each BRCT-Ab2p complex dimer unit was circulated in dashed line. The dimer interface is highlighted in grey and indicated by a black arrow.

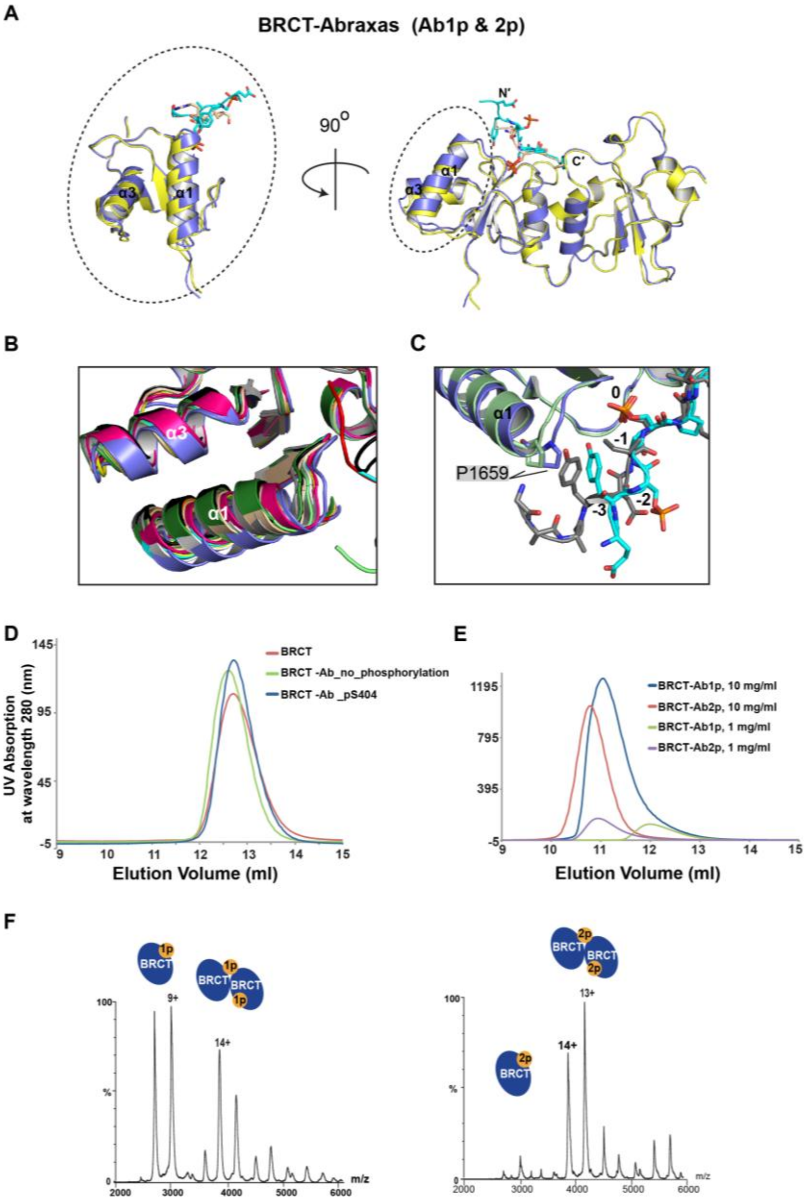

Figure S3 (related to Figure 3): Comparison of BRCT-Abraxas structures with other related BRCA1-BRCT structures, further experiments on the BRCA1-Abraxas complex in solution and native mass spectrometry. A) Superimposition of both BRCT-Ab1p_short (BRCT in yellow and Ab1p_short in wheat colour) and BRCT–Ab2p (BRCT in blue and Ab2p in cyan) structures by aligning the pSPTF motif and the BRCT binding pocket (α2, α1′ and α3′). N- and C-termini are indicated. The black dashed circle highlights the relative structural movements in α1 and α3 and is further zoomed in and rotated 90 degrees. B) Superimposition of BRCT-Ab2p with other BRCT related structures: BRCT-Ab2p (blue), BRCT-Ab1p_short (yellow), BRCT-Bach1 (PDB: 1T15 (cyan) and 1T29 (red) (Clapperton et al., 2004; Shiozaki et al., 2004), BRCT with tetrapeptide motifs (PDB: 3K0K (orange) and 3K0H (wheat)) (Campbell et al., 2010), BRCT with acetyl-coA carboxylase 1 peptide (PDB:3COJ (pink)) (Shen and Tong, 2008) , BRCT-CtIP (PDB: 1Y98 (black)) (Varma et al., 2005), ATRIP (PDB: 4IGK (dark green)) (Liu and Ladias, 2013), optimized peptide (PDB:1T2V (light green)) (Williams et al., 2004) and BAAT1 (PDB:4IFI (white)) (Liu and Ladias, 2013). C) Superimposition of BRCT-Ab2p with BRCT-Optimized peptide (PDB code: 1T2V). The positions of related residues are relative to the phosphorylated serine in pSxxF is in the 0 position. D) Elution profile of BRCT only, BRCT-Ab_no_phosphorylation and BRCT-Ab pS404 are superimposed. BRCT, BRCT-Ab_pS404 and BRCT-Ab_no_phosphorylation are eluted at similar positions. E) BRCT-Ab1p and BRCT-Ab2p at high concentration (10 mg/ml). **(**F) Native mass spectra of BRCT-Abraxas complexes. Protein samples are tested at 75 μM concentration.

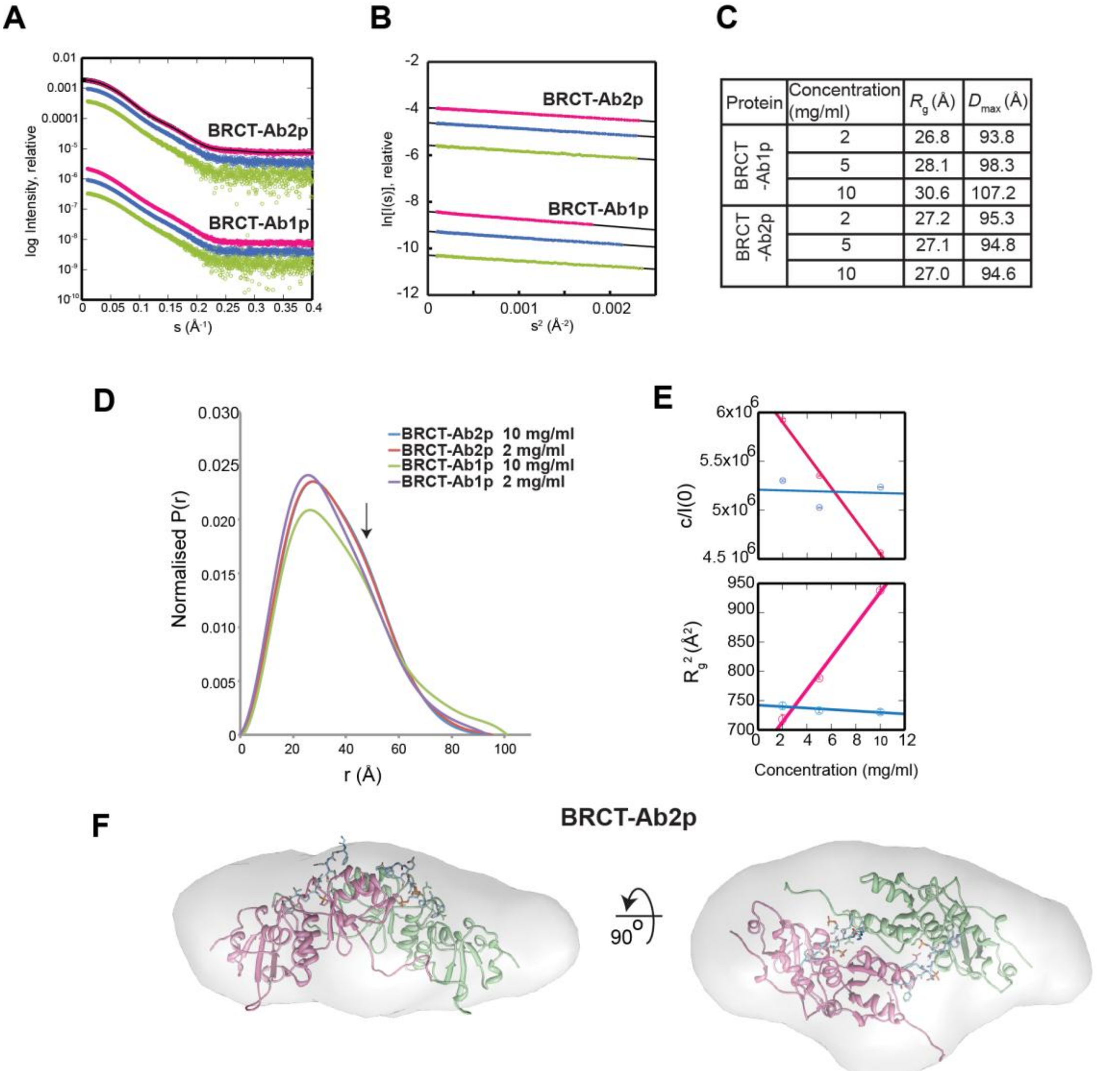

Protein	Concentration (mg/ml)	R_g (Å)	D_{max} (Å)
BRCT -Ab1p	2	26.8	93.8
	5	28.1	98.3
	10	30.6	107.2
BRCT -Ab2p	2	27.2	95.3
	5	27.1	94.8
	10	27.0	94.6

Figure S4 (related to Figure 3). Identification of BRCT-Abraxas using SAXS. A) The SAXS scattering profiles of BRCT-Ab2p at 2 mg/ml (green), 5 mg/ml (blue) and 10 mg/ml (pink) are shifted up compared to those of BRCT-Ab1p for clarity. The black line is simulated-scattering profiles of the crystal structure of BRCT-Ab2p 2:2 dimer complex. B) Guinier plots of BRCT-Ab1p and BRCT-Ab2p. The scattering data (dot; $s<1.3/R_g$) are shown together with linear fit lines (black). C) R_g and D_{max} values of BRCT-Ab1p and BRCT-Ab2p. D) Pairwise distance-distribution functions of BRCT domains. The distribution functions of BRCT-Ab1p at 2 mg/ml (green) and 10 mg/ml (purple) are shown together with that of BRCT-Ab2p 2 mg/ml (red) and BRCT-Ab2p 10 mg/ml (blue). Each distribution function was normalized so that its total area value is 1. E) Concentration dependence of $I(0)$ and radius of gyration R_g of BRCT-Ab1p (pink) and BRCT-Ab2p (blue). The top panel shows the plots of the concentration c versus $c/I(0)$. The lower panel shows the plots of c versus the square of R_g. The top and lower panels share the same abscissa axis. BRCT-Ab1p and BRCT-Ab2p are represented by pink and blue respectively. F) Shape reconstruction of BRCT-Ab2p. The structure of BRCT-Ab2p dimer, of which N-termini were modeled using RapperTK, were fitted in the averaged DAMMIN envelope.

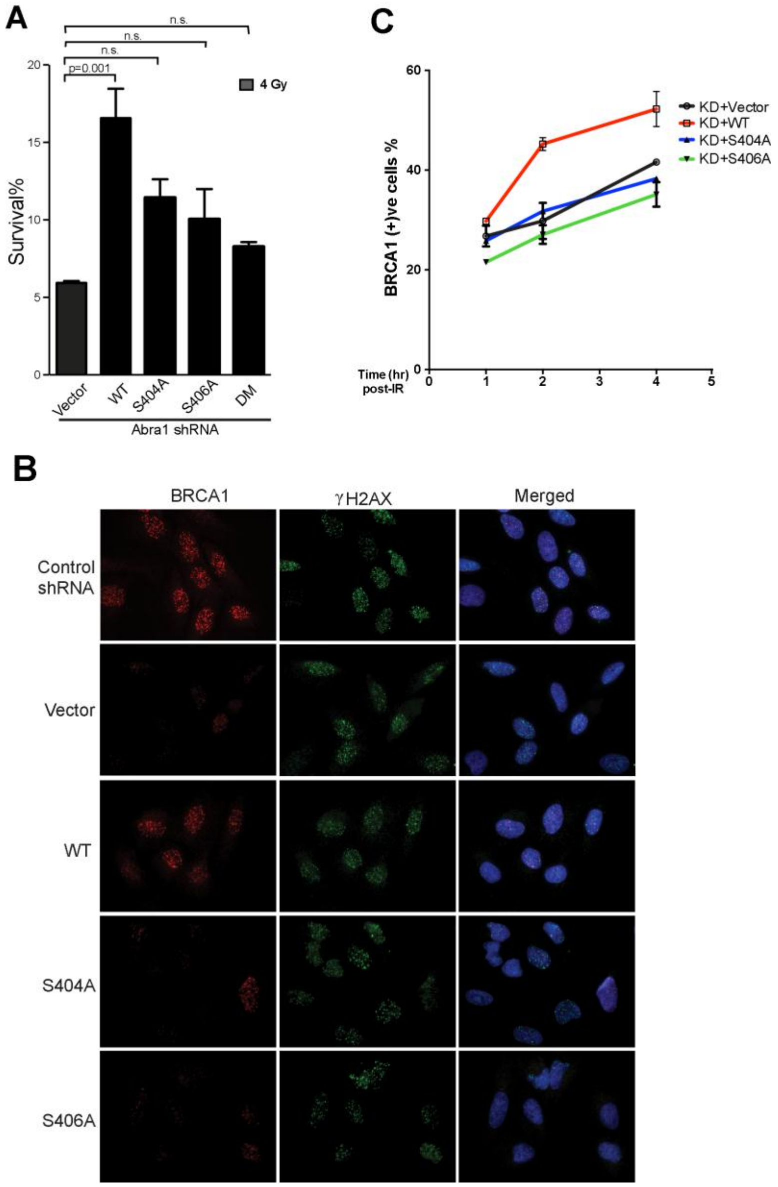

Figure S5 (related to Figure 5): Phosphorylation of S404 and S406 are both important for cellular resistance to IR and BRCA1 accumulation at DNA damage sites. A) An independent colony survival assay for Abraxas-deficient cells expressing WT or mutants of Abraxas treated with 4 Gy IR. Data are presented as means ± s.d. Data analyses are processed by ANOVA and statistical significance was determined by Tukey's multiple comparisons test. B) Representative images of BRCA1 IRIF in Abra1 shRNA knockdown cells complemented with vector, wild-type or mutants of Abraxas in response to 10 Gy IR followed by 2 hr incubation at 37°C. C) Quantification of the percentage of cells containing more than 10 BRCA1 IRIF foci at indicated time points after 10 Gy IR followed by incubation at 37°C. More than 300 cells were counted for each experiment. Data are presented as means ± s.d.

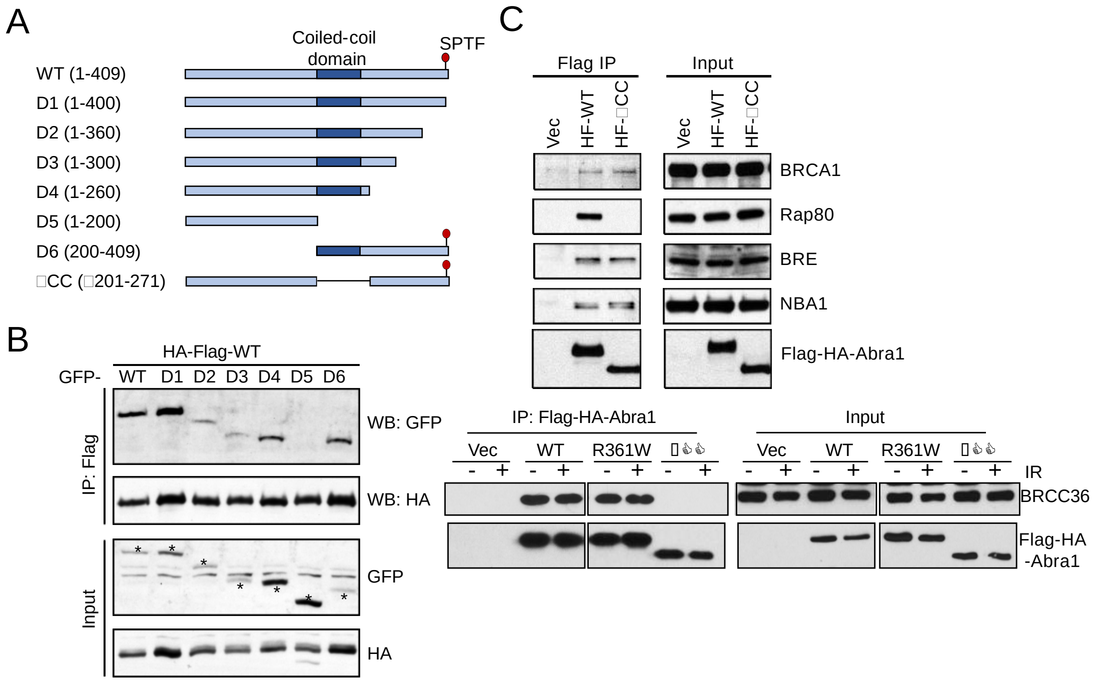

Figure S6 (related to Figure 6): Abraxas forms dimers through the coiled-coil domain independent of binding to BRCA1. A) A schematic view of the Abraxas deletion mutants generated. B) The coiled-coil domain is required for Abraxas dimerization. HA-Flag-tagged wild type (WT) Abraxas were co-transfected with GFP-tagged WT or deletion mutants of Abraxas into 293T cells. 48 h after transfection, cell lysates were prepared and used for Flag-immunoprecipitation. Western blots with GFP or HA antibodies are shown. Expression of GFP-tagged WT or mutant-Abraxas protein products are indicated with “*”. C) Abraxas coiled-coil deletion mutant (ΔCC) retains interaction with other components of the BRCA1-A complex. HA-Flag-tagged WT or ΔCC were transiently expressed in 293T cells. The samples in the upper panel were prepared from cells not treated with ionizing radiation. The lower panel shows the interaction of Abraxas with BRCC36. An Abraxas mutant (R361W) that retains the interaction with BRCC36 was also used as a control. Samples of cells treated with 10 Gy IR or untreated were used to show the interaction of Abraxas with BRCC36. Flag-immunoprecipitation was carried out followed by Western blots with antibodies against indicated protein.

Supplemental Experimental Procedures

Cell Lines, Cell Culture and Antibodies

To generate Abraxas knockdown cells, U2OS cells were infected with retrovirus containing shRNAs against Abraxas followed by selection with puromycin (0.8 ug/ml) for 5 days. The Abraxas knockdown stable cell line was then complemented with expression of empty MSCV vector or expression constructs containing HA-tagged WT or mutant Abraxas, and selected with Blasticidin (9 ug/ml) 1 week for stable expression. 293T Abraxas knockout cells were generated by CRISPR-Cas9 system (Fu et al., 2014; Ran et al., 2013) In brief, 293T cells were infected with lentivirus carrying pLentiCRISPR including Cas9 and sgRNA, which targets Abraxas genomic sequence 5'-GGCGGCGGTAGCATGGA-3'. Cells were then subjected to puromycin selection (2 ug/ml) for three days and plated in low density for culturing for ten days. Single colonies were selected and knockout cells were confirmed by Western blotting with Abraxas antibody and PCR-sequence of the Abraxas genomic locus. Rabbit anti-Abraxas double-phosphorylated pS404pS406 antibody was generated using conjugated GFGEYpSRpSPTF phosphopeptide. The rabbit anti-Abraxas and anti-Abraxas S406 antibodies were generated as previously described (Wang et al., 2007). Other antibodies used were BRCA1 antibodies (D9, Santa Cruz Biotechnologies); GFP antibodies (Invitrogen); γ-tubulin antibodies (Sigma); rabbit anti-HA antibodies (Cell Signaling), Rabbit γH2AX (Upstate), Orc2 (PharMingen).

Immunofluorescence

Abra1 shRNA knockdown cells complemented with empty vector, wild type or mutants of Abra1 were analyzed for BRCA1 IR-induced foci formation (IRIF). At least 500 cells were counted for each cell type and cells containing more than 10 foci were counted as positive. Mean and standard deviations are shown in the Figures 5B,D,E, S5A and statistical analysis was performed by student's t-test or ANOVA with Tukey's multiple comparisons test. p-value is as indicated. All images were obtained with a Nikon TE2000 inverted microscope with a Photometrics Cool- Snap HQ camera. Quantification of BRCA1 was performed using Imaris software (Bitplane). The DAPI channel was used to select the nuclei of the cells in the field, red and green channel were used for BRCA1 and γH2AX, respectively. For BRCA1 foci intensity measurement, foci were defined as particles bigger than 0.25 μM in diameter with an intensity cut-off value (1200) to eliminate background. At least 50 cells were counted and plotted using GraphPad Prism software.

Cell Lysis and Immunoprecipitation

Cells were lysed in NETN buffer (50 mM Tris-HCl, pH 8.0, 150 mM NaCl, 1 mM EDTA, 0.5% Nonidet P-40) with protease inhibitors and protein phosphatase inhibitors, 1 mM NaF, and 1 mM Na_3VO_4. For Flag IP, cell lysates were incubated with Flag beads (Sigma) overnight with gentle agitation at 4^oC. The beads were washed with NETN lysis buffer four times and eluted with 3X sample buffer for Western blot analysis. For analyzing Abraxas dimerization *in vivo*, GFP-tagged and HF-tagged Abraxas wildtype, S404A, S406A mutant or coiled-coil deletion mutant were transiently transfected to 293T cells. Two days after transfection, cells were either untreated or exposed to 10 Gy IR. 2 hr later, cells were collected for GFP- or Flag- IP and Western blot was probed with either antibodies against HA or GFP. For analyzing BRCA1 dimerization *in vivo*, Flag- or Myc-tagged BRCA1 full-length wild type or mutants, or HA- and Myc-tagged BRCA1 BRCT fragments were analyzed in a similar way. ATM inhibitor (KU55933), ATR inhibitor (VE-821), DNA-PK inhibitor (NU7441) and Chk1/2 inhibitor (AZD7762) were purchased from Selleckchem and dissolved in dimethyl sulphoxide (DMSO).

Clonogenic Survival Assay

Abra1 shRNA knockdown cells complemented with empty vector, wild type or mutants of Abra1 were analyzed for cell survival in response to IR. Stable U2OS cell lines were seeded at low density in 10 cm dishes and irradiated with 4 Gy ionizing irradiation using a ^{137}Cs source. The cells were then cultured at 37°C for 14 days to allow colonies to form. Colonies were stained with 2% methylene blue and 50% ethanol for 10 min. Colonies containing 50 or more cells were counted as positive and statistical data were analyzed by analysis of variance (ANOVA) with Tukey's multiple comparisons test.

Chromatin fractionation

Cells were irradiated at 10 Gy followed by 1 hr incubation at 37^oC. For total cell extracts, cells were lysed in NETN150 buffer containing protease inhibitor mixture and analyzed by Western blot. For

chromatin fractionation, irradiated cells were washed in PBS and resuspended in Buffer A (10 mM Hepes pH 7.9, 10 mM KCl, 1.5 mM $MgCl_2$, 0.34 M sucrose, 10% glycerol, 5 mM NaF, 1 mM Na_3VO_4 , 1 mM dithiothreitol (DTT), and protease inhibitor mixture) containing 0.1% Triton X-100, and incubated on ice for 5 min for permeabilization. The cytosolic fraction was then separated by centrifugation at 4000 rpm for 5 min at 4°C. The supernatant was discarded and the nuclei pellet was washed once with Buffer A and resuspended in Buffer B (3 mM EDTA, 0.2 mM EGTA, 1 mM DTT, protease inhibitor mixture) and incubated for 30 min on ice. The soluble nuclear fraction was separated by centrifugation at 4500 rpm for 5 min. The chromatin fraction pellet was washed with Buffer B and resuspended in 100 μl Laemmli sample buffer and sonicated for 10 sec before analysis.

BRCT constructs, expression and purification
The BRCT domains (residues 1646-1859) of the *BRCA1* gene were amplified from IMAGE cDNA (ID 7961446) using PCR and cloned into the pHAT2 vector (with noncleavable N-terminal His-tag) (Peränen et al., 1996) and transformed into Rosetta^{TM}2 (DE3) cells (Invitrogen). Cell culture was grown in LB medium till the OD_{600} was approximately 0.6. IPTG was added to a final concentration of 1 mM and cells were grown overnight at 16°C. 1 g of harvested cells was resuspended with 10 ml of lysis buffer (50 mM Tris pH 8.0, 300 mM NaCl, 20 mM imidazole and one protease inhibitor tablet per 50 ml lysis buffer). Cells were lysed using sonication. The supernatant after centrifugation was then filtered by 0.45 μm filter and loaded onto HisTrap HP 5ml (GE healthcare) pre-equilibrated with binding buffer (50 mM Tris pH 8.0, 300 mM NaCl and 20 mM imidazole) After washing with 10x column volume of binding buffer and binding buffer with 50 mM imidazole, the protein was eluted using elution buffer (50 mM Tris pH 8.0, 300 mM NaCl and 200 mM imidazole). The eluted BRCT domains were about 80% pure as assessed by 12% SDS-PAGE. Remaining contaminants were removed by gel filtration using a Superdex 75 10/300 equilibrated with Buffer A (20 mM Tris pH 8.0, 150 mM NaCl and 5 mM DTT). Fractions containing pure BRCT sample were analyzed in 12% SDS-PAGE gel, pooled, concentrated and stored at -80°C. All mutants were made by site-directed mutagenesis using wt BRCT in pHAT2 as PCR template. Mutant proteins were expressed and purified using the procedure described above.

Phosphopeptides
All phosphopeptides were synthesized to above 95% purity without modification at both N and C terminus (Biomatik). Peptides were dissolved in Buffer A to 25 mg/ml. The pH of the peptide solution was adjusted to near pH 8 using 0.5 M NaOH. The peptide names and sequences are listed below:
Abraxas peptides: 1)Ab1p: GFGEYSRpSPTF; 2)Ab2p: GFGEYpSRpSPTF; 3)Ab1p_short: YSRpSPTF; 4)Ab2p_short: YpSRpSPTF; 5)Ab_pS404: GFGEYpSRSPTF; 6)Ab_no_phosphorylation: GFGEYSRSPTF; 7)Ab2p(Y403A): GFGEApSRpSPTF; 8)Ab2p(Y403A)_short: ApSRpSPTF; 9)Ab2p(F400D): GDGEYpSRpSPTF; 10)Ab2p(E402R): GFGRYpSRpSPTF; 11)Ab1p(S404P): GFGEYPRpSPTF; 12)Ab1p(S404D): GFGEYDRpSPTF; 13)Bach1: ISRSTpSPTFNKQ; 14) CtIP: PTRVSpSPVFGAT

Protein crystallization and data collection
Crystals were cryoprotected in crystallization solution containing 30% glycerol using the two-step transferring method and then subsequently flash frozen in liquid nitrogen. The structure of BRCT-Bach1 (PDB code 1T15) (Clapperton et al., 2004) without Bach1 phosphopeptide was used as the search model. Relaxed NCS restraints among all the molecules of the BRCT-Ab2p in the asymmetric unit were used in the refinement protocol as defined in phaser_refine module. This resulted in clear $2F_o$-F_c and F_o-F_c electron density that allowed manual building of the Abraxas peptide using *Coot* (Emsley et al., 2010) (Figure S1). For BRCT-Ab2p, the structure connectivity and disordered region vary among molecules in the ASU depending on its position in the crystallographic packing, therefore chains IDs G, H, O and P have poorer electron density map than other chains. Further structure refinements also included simulated annealing, optimizing X-ray/Stereochemistry weight, optimizing X-ray/ADP weight and side chain adjustment using *Coot*. The buried surface area after dimerization of BRCT-Ab2p was calculated using PISA (Krissinel and Henrick, 2007).

Identification of BRCT-Ab1p and BRCT-Ab2p using SAXS
We carried out SAXS of the BRCT-Ab1p and BRCT-Ab2p in different concentrations. The scattering profiles (Figure S4A) of BRCT-Ab1p and BRCT-Ab2p at high concentration (10 mg/ml) were similar and indicated that the samples were monodisperse (Figure S4B). The scattering profiles of BRCT-Ab2p did not change by increasing the concentration of the sample whereas BRCT-Ab1p changed particularly at low angle. We then used the BRCT-Ab2p dimer crystal structure to calculate its

scattering profile and compare it with the observed profile of BRCT-Ab2p (black line in Figure S4A). The calculated-scattering profiles of the 2:2 BRCT-Ab2p dimer explain the observed scattering profiles much better than for the 1:1 complex (χ=1.675 and 3.806 respectively) indicating that BRCT-Ab2p is dimerized in solution. The fitting of the 2:2 dimer structure against the scattering profile of BRCT-Ab1p was better at 10 mg/ml (χ=1.934) than at 2 mg/ml (χ=5.975). The pairwise distance-distribution functions of BRCT-Abraxas complexes showed that BRCT-Ab2p has a very similar profile at both 10 mg/ml and 2 mg/ml concentrations. They both have a shoulder around 50 Å (indicated by black arrow in Figure S4D), which is clearer than that of BRCT-Ab1p at 10 mg/ml and 2 mg/ml. Furthermore, BRCT-Ab1p showed inter-particle attraction by increasing the concentration of the sample but BRCT-Ab2p did not (Figure S4E) (Nakasako et al., 2005). BRCT-Ab2p complex dimer crystal structure can be fitted into the envelope of an averaged DAMMIN model of the dimer (Figure S4F).

SAXS analysis
SAXS data of 2, 5 and 10 mg/ml BRCT-Ab1p and BRCT-Ab2p in Buffer A (20 mM Tris pH 8.0, 150 mM NaCl and 5 mM DTT) were collected at I22 in Diamond Light Source. Protein samples were loaded into glass capillaries using a BIOSAXS robot and the scattering intensities of the sample were measured at 5°C using the Pilatus 2M detector. The scattering vector s is $4\pi\sin\theta/\lambda$, where θ is half of the scattering angle and λ is the wavelength (0.9987 Å). Data reduction was carried out using DAWN. Primus was used to interpolate to the zero concentration of BRCT-Abraxas2p data using the ZeroConc module and to calculate R_g & $I(0)$ using the AutoRg module and pair-distribution functions & $D_{\max}$ using the AutoGNOM module (Figure S4C) (Petoukhov et al., 2012). Ten dummy-residue models of BRCT-Ab2p were created using DAMMIN (Svergun, 1999) and were superimposed and averaged using SUPCOMB (Kozin and Svergun, 2001) and DAMAVER (Volkov and Svergun, 2003) to generate a dummy-residue model of BRCT-Ab2p. The mean value of normalized-spatial discrepancy was 0.567. The selection of the best-fit model was conducted as follows. The missing N-termini of the BRCT domains and the peptide from BRCT-Ab2p crystal structure were added by *Coot* (Emsley et al., 2010). RapperTK (Gore et al., 2007) was used to generated 100 conformations of these flexible structures. A conformation of each chain was randomly selected and mixed to generate 1000 conformations of BRCT-Ab2p. The fitting of each conformation was calculated using CRYSOL to select the best conformation.

Supplemental References

Campbell, S.J., Edwards, R.A., and Glover, J.N.M. (2010). Comparison of the structures and peptide binding specificities of the BRCT domains of MDC1 and BRCA1. Structure *18*, 167–176.

Clapperton, J.A., Manke, I.A., Lowery, D.M., Ho, T., Haire, L.F., Yaffe, M.B., and Smerdon, S.J. (2004). Structure and mechanism of BRCA1 BRCT domain recognition of phosphorylated BACH1 with implications for cancer. Nat. Struct. Mol. Biol. *11*, 512–518.

Emsley, P., Lohkamp, B., Scott, W.G., and Cowtan, K. (2010). Features and development of Coot. Acta Crystallogr. D. Biol. Crystallogr. *66*, 486–501.

Fu, Y., Sander, J.D., Reyon, D., Cascio, V.M., and Joung, J.K. (2014). Improving CRISPR-Cas nuclease specificity using truncated guide RNAs. Nat Biotech *32*, 279–284.

Gore, S.P., Karmali, A.M., and Blundell, T.L. (2007). Rappertk: a versatile engine for discrete restraint-based conformational sampling of macromolecules. BMC Struct. Biol. *7*.

Kozin, M.B., and Svergun, D.I. (2001). Automated matching of high- and low-resolution structural models. J. Appl. Crystallogr. *34*, 33–41.

Krissinel, E., and Henrick, K. (2007). Inference of macromolecular assemblies from crystalline state. J. Mol. Biol. *372*, 774–797.

Liu, X., and Ladias, J.A.A. (2013). Structural basis for the BRCA1 BRCT interaction with the proteins ATRIP and BAAT1. Biochemistry *52*, 7618–7627.

Nakasako, M., Iwata, T., Inoue, K., and Tokutomi, S. (2005). Light-induced global structural changes in phytochrome A regulating photomorphogenesis in plants. FEBS J. *272*, 603–612.

Peränen, J., Rikkonen, M., Hyvönen, M., and Kääriäinen, L. (1996). T7 vectors with modified T7lac promoter for expression of proteins in Escherichia coli. Anal. Biochem. *236*, 371–373.

Petoukhov, M. V., Franke, D., Shkumatov, A. V., Tria, G., Kikhney, A.G., Gajda, M., Gorba, C., Mertens, H.D.T., Konarev, P. V., and Svergun, D.I. (2012). New developments in the ATSAS program package for small-angle scattering data analysis. J. Appl. Crystallogr. *45*, 342–350.

Ran, F.A., Hsu, P.D., Wright, J., Agarwala, V., Scott, D.A., and Zhang, F. (2013). Genome engineering using the CRISPR-Cas9 system. Nat. Protoc. *8*, 2281–2308.

Shen, Y., and Tong, L. (2008). Structural evidence for direct interactions between the BRCT domains of human BRCA1 and a phospho-peptide from human ACC1. Biochemistry *47*, 5767–5773.

Shiozaki, E.N., Gu, L., Yan, N., and Shi, Y. (2004). Structure of the BRCT repeats of BRCA1 bound to a BACH1 phosphopeptide: implications for signaling. Mol. Cell *14*, 405–412.

Svergun, D.I. (1999). Restoring Low Resolution Structure of Biological Macromolecules from Solution Scattering Using Simulated Annealing. *76*, 2879–2886.

Varma, A.K., Brown, R.S., Birrane, G., and Ladias, J.A.A. (2005). Structural Basis for Cell Cycle Checkpoint Control by the BRCA1−CtIP Complex. Biochemistry *44*, 10941–10946.

Volkov, V. V, and Svergun, D.I. (2003). Uniqueness of ab initio shape determination in small-angle scattering. J. Appl. Crystallogr. *36*, 860–864.

Wang, B., Matsuoka, S., Ballif, B.A., Zhang, D., Smogorzewska, A., Gygi, S.P., and Elledge, S.J. (2007). Abraxas and RAP80 Form a BRCA1 Protein Complex Required for the DNA Damage Response. Science (80-.). *316*, 1194–1198.

Williams, R.S., Lee, M.S., Hau, D.D., and Glover, J.N.M. (2004). Structural basis of phosphopeptide recognition by the BRCT domain of BRCA1. Nat Struct Mol Biol *11*, 519–525.
