# Supplementary material for: Structure of BRCA1-BRCT/Abraxas Complex Reveals Phosphorylation-Dependent BRCT Dimerization at DNA Damage Sites
Source: Mol Cell. 2016 Feb 4;61(3):434–48. doi: 10.1016/j.molcel.2015.12.017 (PMC4747905; doi:10.1016/j.molcel.2015.12.017)
Supplement: Document S2. Article plus Supplemental Information [file mmc2.pdf]

Molecular Cell

Article

Structure of BRCA1-BRCT/Abraxas Complex Reveals Phosphorylation-Dependent BRCT Dimerization at DNA Damage Sites

Graphical Abstract

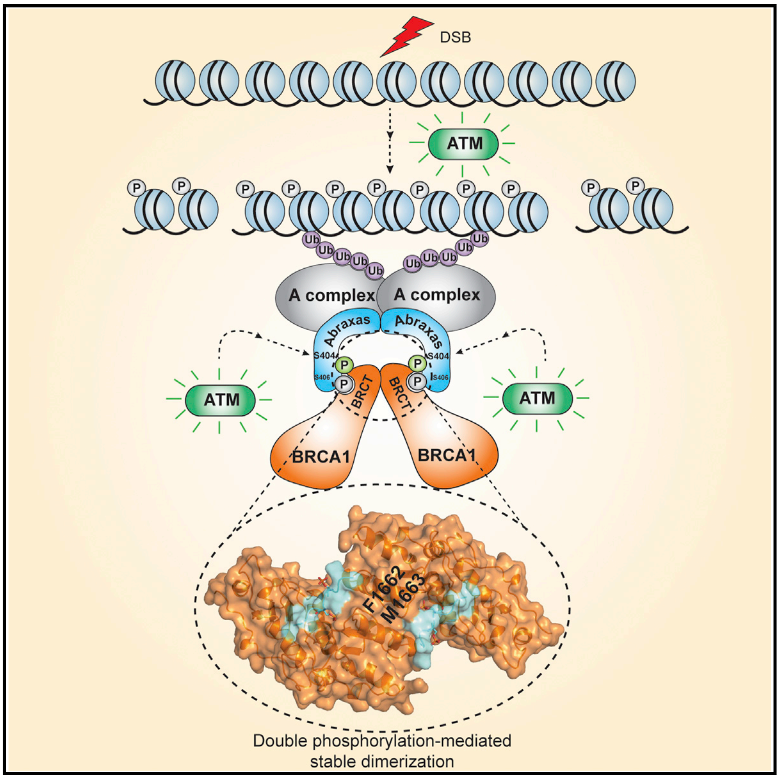

Authors

Qian Wu, Atanu Paul, Dan Su, ...,
Carol V. Robinson, Bin Wang,
Tom L. Blundell

Correspondence

bwang3@mdanderson.org (B.W.),
tom@cryst.bioc.cam.ac.uk (T.L.B.)

In Brief

Abraxas interacts with BRCA1 through a phosphor-SPxF motif. Wu et al. show that a nearby serine 404 residue is phosphorylated in response to ionizing radiation in an ATM-dependent manner. S404 phosphorylation promotes BRCA1-BRCT/Abraxas dimer formation and is crucial for BRCA1 accumulation at DNA damage sites.

Highlights

- Abraxas C terminus S404 is phosphorylated in response to ionizing radiation
- Phosphorylated S404 promotes stable BRCA1 BRCT/ Abraxas dimer formation
- Abraxas S404 is critical for BRCA1 accumulation at DNA damage sites
- BRCA1 germline mutations disrupt BRCA1-BRCT/Abraxas dimer formation

Accession Numbers

4Y2G
4Y18

Wu et al., 2016, Molecular Cell *61*, 434–448
February 4, 2016 ©2016 The Authors
http://dx.doi.org/10.1016/j.molcel.2015.12.017

CellPress

Molecular Cell
Article

Structure of BRCA1-BRCT/Abraxas Complex Reveals Phosphorylation-Dependent BRCT Dimerization at DNA Damage Sites

Qian Wu,[1,6] Atanu Paul,[2,3,6] Dan Su,[2] Shahid Mehmood,[4] Tzeh Keong Foo,[5] Takashi Ochi,[1] Emma L. Bunting,[1] Bing Xia,[5] Carol V. Robinson,[4] Bin Wang,[2,3,*] and Tom L. Blundell[1,*]
[1]Department of Biochemistry, University of Cambridge, 80 Tennis Court Road, CB2 1GA Cambridge, UK
[2]Department of Genetics, The University of Texas MD Anderson Cancer Center, 1515 Holcombe Boulevard, Houston, TX 77030, USA
[3]Genes and Development Program, The University of Texas Graduate School of Biomedical Sciences at Houston, 6767 Bertner Avenue, Houston, TX 77030, USA
[4]Department of Chemistry, Physical and Theoretical Chemistry Laboratory, University of Oxford, South Parks Road, OX1 3QZ Oxford, UK
[5]Department of Radiation Oncology, Robert Wood Johnson Medical School, Rutgers Cancer Institute of New Jersey, 195 Little Albany Street, New Brunswick, NJ 08903, USA
[6]Co-first author
*Correspondence: bwang3@mdanderson.org (B.W.), tom@cryst.bioc.cam.ac.uk (T.L.B.)
http://dx.doi.org/10.1016/j.molcel.2015.12.017
This is an open access article under the CC BY license (http://creativecommons.org/licenses/by/4.0/).

SUMMARY

BRCA1 accumulation at DNA damage sites is an important step for its function in the DNA damage response and in DNA repair. BRCA1-BRCT domains bind to proteins containing the phosphorylated serine-proline-x-phenylalanine (pSPxF) motif including Abraxas, Bach1/FancJ, and CtIP. In this study, we demonstrate that ionizing radiation (IR)-induces ATM-dependent phosphorylation of serine 404 (S404) next to the pSPxF motif. Crystal structures of BRCT/Abraxas show that phosphorylation of S404 is important for extensive interactions through the N-terminal sequence outside the pSPxF motif and leads to formation of a stable dimer. Mutation of S404 leads to deficiency in BRCA1 accumulation at DNA damage sites and cellular sensitivity to IR. In addition, two germline mutations of BRCA1 are found to disrupt the dimer interface and dimer formation. Thus, we demonstrate a mechanism involving IR-induced phosphorylation and dimerization of the BRCT/Abraxas complex for regulating Abraxas-mediated recruitment of BRCA1 in response to IR.

INTRODUCTION

Patients with hereditary breast and ovarian cancer (HBOC) have high germline gene-mutation rates on chromosome 17q21 tumor suppressor gene *BRCA1* (breast cancer susceptibility genes 1) (Futreal et al., 1994; Hall et al., 1990; Miki et al., 1994). BRCA1 stabilizes genomic integrity by interacting with various DNA damage response (DDR) sensors, mediators, and effector proteins, thereby coordinating recognition of the DNA damage sites, cell-cycle checkpoint, DNA repair, transcription, and apoptosis/senescence. BRCA1, a large protein of 1,863 amino acids, contains an N-terminal RING domain and two C-terminal tandem BRCT domains. BRCT domains can recognize phosphorylated proteins with a phosphorylated serine-proline-x-phenylalanine (pSPxF) motif (Manke et al., 2003; Rodriguez et al., 2003; Yu et al., 2003) including Abraxas (Kim et al., 2007a; Liu et al., 2007; Wang et al., 2007), Bach1/FancJ (Cantor et al., 2001; Yu et al., 2003), and CtIP (Wong et al., 1998; Yu et al., 1998). The phosphopeptide-binding ability of BRCA1 BRCT is essential for BRCA1's tumor suppression function (Shakya et al., 2011), where many breast and ovarian cancer related mutations occur (Clapperton et al., 2004; Couch and Weber, 1996; Friedman et al., 1994; Shattuck-Eidens et al., 1995; Shiozaki et al., 2004; Williams et al., 2004).

The two BRCA1-BRCT domains (BRCT1 and BRCT2) each contain about 100 residues and associate in a head-to-tail manner (Williams et al., 2001). Structural analysis of BRCA1-BRCT domains with pSPxF-containing phosphopeptides of Bach1 (Clapperton et al., 2004; Shiozaki et al., 2004), CtIP (Varma et al., 2005), synthetic optimized phosphopetide (Williams et al., 2004), or other binding proteins (Campbell et al., 2010; Liu and Ladias, 2013; Shen and Tong, 2008) have revealed that phosphorylated serine and phenylalanine in the pSPxF motif bind in a cleft formed at the junction of two BRCT domains in a "two-anchor" mode and the structural integrity of both binding sites is essential for peptide recognition (Glover et al., 2004; Leung and Glover, 2011; Wu et al., 2015). However, little information exists regarding the importance of the sequence surrounding the pSPxF motif. Nor is it known how the specificity is determined for BRCT binding to different pSPxF motif-containing proteins.

Abraxas mediates the interaction of BRCA1 to other components of the BRCA1-A complex, which include BRCC36, NBA1/MERIT40, BRE, and Rap80. Abraxas, a 409-residue polypeptide, contains a non-catalytic Mpr1, Pad1 N-terminal (MPN)

domain at its N terminus, followed by a coiled-coil (CC) region, an unstructured region, and a BRCA1-binding pSPTF motif at the C terminus. While the N-terminal region including the MPN domain binds to Rap80, BRE, and NBA1/MERIT40, the CC domain is required for interaction with BRCC36 (Hu et al., 2011; Kim et al., 2007a; Wang and Elledge, 2007; Wang et al., 2009). Although the structures for BRCA1 BRCT in complex with other phosphopeptides have been solved previously (Campbell et al., 2010; Clapperton et al., 2004; Liu and Ladias, 2013; Shen and Tong, 2008; Shiozaki et al., 2004; Varma et al., 2005; Williams et al., 2004), the structure for BRCT/Abraxas has remained unknown.

Abraxas and the BRCA1-A complex recruit BRCA1 to DNA double-strand-break sites (DSBs) in an ATM-dependent ubiquitin-mediated signaling pathway involving E2 conjugase Ubc13, E3 ligases RNF8/RNF168, and Rap80 binding to ubiquitin lys63-linked polyubiquitin conjugates (Doil et al., 2009; Harper and Elledge, 2007; Hu et al., 2012; Huen and Chen, 2008; Kim et al., 2007b; Kolas et al., 2007; Lee and Paull, 2004; Mailand et al., 2007; Sato et al., 2009; Uziel et al., 2003; Wang, 2012; Wang and Elledge, 2007; Wu et al., 2009). Abraxas-deficient mice exhibit decreased survival and increased tumor incidence (Castillo et al., 2014). The interaction of Abraxas with BRCA1 has been shown critical for the function of Abraxas in DNA repair of DSBs and maintenance of genomic stability. Mutation of the serine residue in the pSPxF motif leads to defective DNA repair and chromosomal aberration. The importance of the Abraxas-BRCA1 interaction in tumor suppression is also suggested by identification of an Abraxas mutation in tumor in the phenylalanine residue of the pSPxF motif (F409C) (Castillo et al., 2014). Thus, structural and functional analysis of Abraxas and BRCA1 interaction is necessary to facilitate the understanding of Abraxas-mediated BRCA1 signaling in tumor suppression.

In this study, we have solved the crystal structures of BRCT with Abraxas phosphorylated peptides and revealed an ionizing radiation (IR)-induced, ATM-dependent Abraxas phosphorylation mechanism, which promotes dimerization of BRCT/Abraxas complex at the DNA damage sites. The IR-induced phosphorylation of Abraxas and the subsequent stabilization of BRCA1-BRCT dimerization are likely to comprise an important mechanism for accumulation of BRCA1 to DNA-damaged chromatin and BRCA1 mediated tumor suppression.

RESULTS

Abraxas Is Double Phosphorylated at S404 and S406 Residues in Response to IR

Analysis of the C-terminal sequence of Abraxas reveals an additional serine (S404) located close to the pSPxF motif (Figure 1A). Double-phosphorylated Abraxas peptide containing phosphorylated S406 and S404 (GFGEYpS^{404}RpS^{406}PTF) has been identified to bind to BRCA1-BRCT domains in response to IR (Wang et al., 2007). We decided to investigate whether the S404 residue is important and whether it is phosphorylated upon IR. Previously, we have generated S406 phospho-specific antibody and showed that phosphorylation of S406 (pS406) occurs independently of IR (Wang et al., 2007). In view of the fact that S406 is nearby and is phosphorylated in the presence and absence of IR, we generated antibodies specifically recognizing double phosphorylated S404 and S406 (pS404pS406). The pS404pS406 specific antibody recognized Abraxas in parental, but not Abraxas knockout 293T cells and the intensity of the Abraxas band increased significantly when cells were treated with IR, indicative of IR-induced phosphorylation (Figure 1B). Mutation of either S404 (S404A), S406 (S406A), or double mutation (S404A and S406A) abolished the recognition of Abraxas by the pS404pS406 antibody (Figure 1C). Upon IR treatment, double phosphorylation of Abraxas S404 and S406 residues increased immediately (within 10 min), peaked at 1 hr, and gradually decreased to nearly basal levels at later time points (Figure 1D). Furthermore, double phosphorylation occurs in a dose-dependent manner in response to IR (Figure 1E). Since phosphorylation of S406 is not changed upon IR treatment, phosphorylation of S404 is likely to be IR-induced. As ATM plays a central role in the IR-induced signaling pathway that recruits Abraxas and the BRCA1-A complex (Harper and Elledge, 2007), we investigated whether ATM regulates the phosphorylation. Indeed, the IR-induced S404 phosphorylation is ATM dependent, treatment of an ATM inhibitor KU55933 completely abolished the IR-induced phosphorylation (Figure 1F). Other DDR kinases including ATR, DNA-PK, Chk1, or Chk2, however, did not appear to have a major effect on the IR-induced phosphorylation recognized by the pS404pS406 antibody (Figures 1G and S1).

Crystal Structures of BRCA1 BRCT Domains in Complex with Single and Double-Phosphorylated Abraxas Peptides

Since S404 is separated by just one residue from S406, which is part of the pSPxF motif, we hypothesized that an additional mechanism exploiting phosphorylation of S404 might regulate Abraxas interaction with BRCA1. In order to test this, we used structural information to allow comparison of the interactions between BRCT and Abraxas in single and double-phosphorylated states. We solved crystal structures of BRCA1-BRCT in complex with both single (1p) and double phosphorylated (2p) synthesized Abraxas peptides (Ab). The BRCT-Ab1p (GFGEYSRpSPTF) complex crystal was solved at 3.5 Å resolution with no clear main-chain electron density for the N-terminal GFGE region of the peptide, indicating that this region is flexible. BRCT-Ab1p_short without the GFGE residues (YSRpSPTF) complex was then crystalized and solved at 2.5 Å resolution. The structure of BRCT domains in complex with the 2p Ab (Ab2p: GFGEYpSRpSPTF) was solved at 3.5 Å resolution. However, the BRCT-Ab2p_short (YpSRpSPTF) crystal did not diffract to a high resolution. We therefore used BRCT-Ab1p_short and BRCT-Ab2p structures (Figures 2A and S2A) for analysis. Statistics of these two structures are shown in Table 1.

As in structures solved previously (Campbell et al., 2010; Clapperton et al., 2004; Liu and Ladias, 2013; Shen and Tong, 2008; Shiozaki et al., 2004; Varma et al., 2005; Williams et al., 2001, 2004), two BRCT domains of BRCA1 (BRCT1 and BRCT2) associate in a head-to-tail manner in both structures. In each domain, a four-stranded parallel β sheet is surrounded by three α helices with α1 and α3 on one side of the β sheet and α2 on the other side. Helices α2 (from BRCT1), α′1, and α′3 (from BRCT2) form

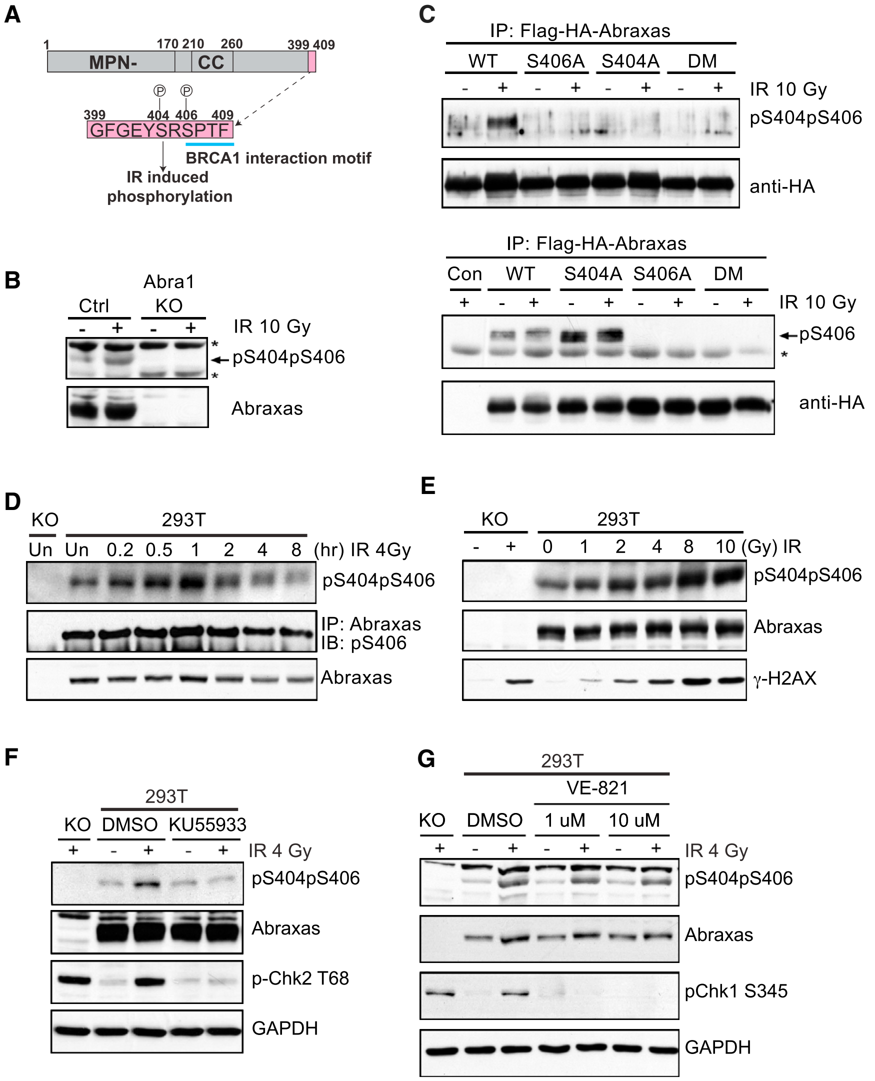

Figure 1. IR-Induced Double Phosphorylation of Abraxas C Terminus S404 and S406 Is ATM Dependent

(A) Abraxas-domain boundary and C-terminal sequence containing a serine residue (S404) next to the BRCA1-binding pSPxF motif (high-lighted in blue). The phosphorylation of S404 and S406 is indicated as P.

(B) Double phosphorylation of S404 and S406 residues at the Abraxas C terminus in response to IR in 293T cells and 293T/Abraxas KO cells. The lysates from cells treated with 10 Gy IR followed by incubation at 37°C for 1 hr were used for western blot with anti-pS404pS406 antibody ("*" non-specific band).

(C) IR-induced double phosphorylation of S404 and S406 is abolished in Abraxas mutants (S404A, S406A, or double mutant, DM) ("*" non-specific band). The FLAG- and HA-tagged Abraxas WT or mutants were expressed in 293T cells. The lysates from cells treated with 10 Gy IR and incubated at 37°C for 1 hr were used for immunoprecipitation with anti-FLAG beads and western blot with antibodies against pS404pS406, pS406, or HA.

(D) IR-induced double phosphorylation of S404 and S406 occurs immediately after IR treatment. The time points were taken after cells were treated with 4 Gy IR followed by incubation at 37°C.

(E) IR-induced phosphorylation occurs in a dose-dependent manner.

(F) ATM regulates IR-induced phosphorylation. The cells were incubated with ATM kinase inhibitor KU55933 (10 μM) for 2 hr before exposure to 4 Gy IR and subsequent incubation at 37°C for 1 hr.

(G) ATR is not involved in IR-induced double phosphorylation. The ATR inhibitor VE-821 at indicated concentrations was used for treating cells for 2 hr before cells were exposed to 4 Gy IR (see also Figure S1).

the hydrophobic interface, and the two domains are further linked by extra helix αL (Figures 2A and 2E). The pSPTF motif from Abraxas binds to the BRCT domains in a similar two-anchor mode using pS406 and F409. Residues P407 and T408 do not make major interactions with the BRCT domains. The phosphate group of Abraxas S406 interacts with the side chains of BRCT K1702 and S1655, as well as the main chain of G1656 (Figure 2B). The side chain of F409 in Abraxas inserts into the BRCT hydrophobic pocket created by L1701, F1704, N1774, M1775, and L1839 (Figure 2C). As F409 is the terminal residue for Abraxas, an extra salt bridge is present between the main chain carboxyl group of F409 with the BRCT domain residue R1699 (Figure 2D). This extra interaction was seen in previous structures using tetrapeptides pSPTF (Campbell et al., 2010).

A notable difference between BRCT-Ab2p and BRCT-Ab1p_short structure is the conformation of the $Y^{403}S^{404}R^{405}$ region (Figures 2E–2G). Extra electron density corresponding to the phosphate group of pS404 and the side chain of Y403 is observed only in BRCT-Ab2p. Unlike pS406, the pS404 phosphate group is oriented away from the BRCT domains into the solvent region, thus avoiding contact with G1656, L1657, and T1658 (Figure 2G). In BRCT-Ab2p, the Y403 side chain is positioned to interact through a hydrophobic interaction with BRCT P1659 at the N terminus of BRCT1 α1. The extra interaction could explain the increased proximity of α1 toward the N terminus of the Abraxas phosphopeptide in BRCT-Ab2p compared to BRCT-Ab1p_short (Figure S3A). Superimposition of all available BRCA1 BRCT related crystal structures also showed that α1 movement toward the phosphopeptide is most prominent in BRCT-Ab2p (Figure S2B). It indicates that one of the roles of pS404 is to fix the side chain of Y403, which is conserved in higher organisms (Figure 2I), such that a *trans* peptide bond can form and collision is avoided. Superimposition of the BRCT/Abraxas structures with the BRCT/Bach1 (PDB: 1T29) (Shiozaki et al., 2004) and BRCT/CtIP structures (PDB: 1Y98) (Varma et al., 2005) shows similar pSPxF-motif binding. However, compared to Bach1 and CtIP, the N-terminal sequence of Abraxas in both BRCT-Ab1p and BRCT-Ab2p structures exits

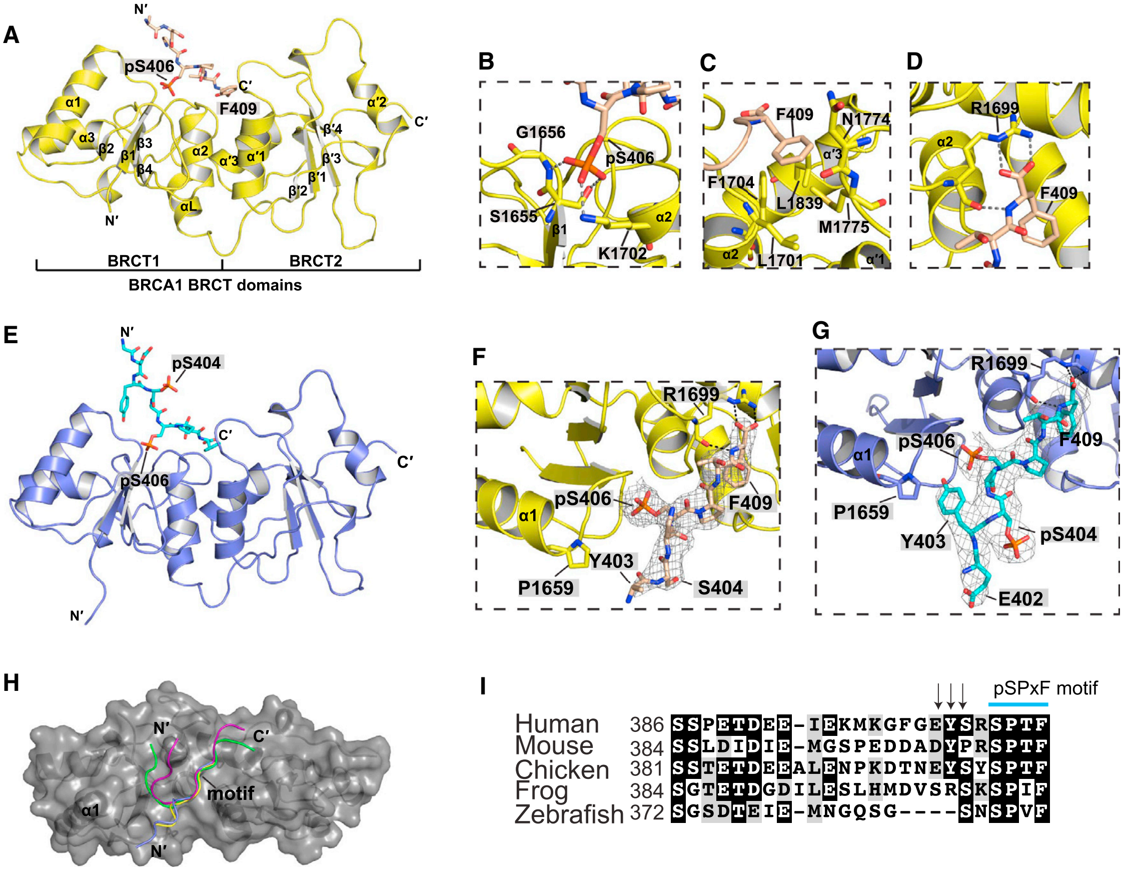

Figure 2. Crystal Structures of BRCT in Complex with Single and Double Phosphorylated Abraxas Peptide

(A) Crystal structure of BRCT-Ab1p_short. The BRCT domains are in yellow, and the Ab1p_short peptide is in wheat color.

(B–D) Show the detailed interactions between phosphopeptide and BRCT domains. The polar interaction is indicated in dashed lines.

(E) Crystal structure of BRCT-Ab2p. The BRCT is in blue, and the Ab2p peptide is in cyan.

(F and G) Show the interface between BRCT and Ab in both BRCT-Ab1p_short and BRCT-Ab2p structures. The *2Fo-Fc* electron density ($\sigma = 1.0$) is shown for Abs.

(H) Superimposition of BRCT-Ab2p, BRCT-Ab1p_short, BRCT-Bach1 (PDB code: 1T29), and BRCT-CtIP (PDB code: 1Y98). The BRCT domains are shown in a gray surface representation. Ab2p, Ab1p_short, Bach1, and CtIP are in blue, yellow, green, and purple, respectively. The pSPxF motif is indicated in the image.

(I) Sequence alignment of Abraxas C terminus. The BRCT-binding motif is indicated by a blue line, and the black arrows indicate the half conserved residues (see also Figures S2 and S3).

on the opposite side, close to the α1 of BRCT1 domain (Figure 2H). Interestingly, a similar side chain arrangement was also seen in BRCA1 BRCT bound with "optimized peptide" (GAAYDIpSQVFPFAKKK) (PDB: 1T2V) (Williams et al., 2004) (Figure S3C), in which the tyrosine residue (Y) at -3 position (phosphorylated serine in pSxxF motif as 0 position) and negatively charged residues glutamic acid (E) or aspartic acid (D) at -2 position were shown more favored for interaction with BRCA1 BRCT domains (Manke et al., 2003; Rodriguez et al., 2003).

Dimerization of BRCT-Abraxas Complex in Crystal Structures

There are eight copies of BRCT-Ab2p in the asymmetric unit (ASU), and each BRCT-Ab2p appears to dimerize through the same interface either within the ASU or between ASUs (Figure S2B). The dimerization of the 1:1 BRCT-Ab2p complex results in a 2:2 BRCT/Abraxas complex dimer. Dimerization involves α1 and β2 of the BRCT1 domain and the Ab2p (Figures 3A–3D) burying about 1,880 $Å^2$ area. In the dimer interface, two of the α1 helices from each BRCT-Ab2p complex form isologous interactions burying a hydrophobic patch formed by F1662, M1663, and Y1666 with aromatic side chains stacking on each other (Figure 3B). Interestingly, BRCA1 germline mutations of F1662 (F1662S) and M1663 (M1663K) have been identified in germline cancer patients as recorded in the Breast Cancer Information Core database (Szabo et al., 2000). Extensive hydrogen bonds also form between the two equivalent antiparallel β2 strands (residues $T^{1,675}$–$L^{1,679}$) (Figure 3D). The two-fold symmetry axis within the BRCA1/Abraxas dimer lies perpendicular to the two β strands. The cross interaction between the two BRCT/Abraxas complexes is also mediated by the ionic interaction between Abraxas and BRCT α1 of the opposite BRCT/Abraxas complex. The negative surface patch, generated by the phosphate group of pS404 and side chain of E402 at the N terminus of Ab2p peptide, leads to cross interaction with

Table 1. The Statistics of Structures

Crystals	BRCT-Ab1p_short	BRCT-Ab2p
X-ray source	Diamond Light Source Beamline I03	Diamond Light Source Beamline I04-1
Wavelength (Å)	0.9793	0.9200
Space group	$P3_221$	$P2_12_12_1$
Cell dimensions a, b, c (Å) and α, β, γ (°)	63.8, 63.8, 93.4, 90, 90, and 120	86.8, 183.7, 190.5, 90, 90, and 90
Resolution (Å)	47.6-2.5	95.3-3.5
R_{sym}[a]	0.051 (0.498)[b]	0.096 (0.701)
I / σ	24 (5.1)	16.4 (2.6)
Wilson B factor	58.6	97.2
Completeness (%)	99.9 (99.8)	99.8 (98.8)
Redundancy	9.3 (9.4)	7.4 (7.8)
Refinement		
Resolution (Å)	47.6-2.5 (2.6-2.5)	95.3-3.5 (3.7-3.5)
No. unique reflections	8,000	39,170
R_{cryst}[c]	0.215 (0.356)	0.235 (0.319)
R_{free}[d]	0.252 (0.374)	0.298 (0.341)
No. protein atoms	1,701	14,092
No. copy number of complex in ASU	1	8
Ramachandran favored (%)	97.7	97.1
Average B-factor (Å^2)	66.68	114.6
Rmsds		
Bond lengths (Å)	0.007	0.007
Bond angles (°)	0.823	0.900

[a]$R_{sym} = \Sigma_h|I_h - <I>|/\Sigma_h I_h$, where I_h is the intensity of reflection h and $<I>$ is the mean intensity of all symmetry-related reflections.
[b]The statistics in parenthesis are for the highest resolution shell.
[c]$R_{cryst} = \Sigma||F_{obs}|-|F_{calc}||/\Sigma|F_{obs}|$, F_{obs} and F_{calc} are observed and calculated structure factor amplitudes.
[d]R_{free} as for R_{cryst} using a random subset of the data (about 10% for BRCT-Ab1p_short and 5% for BRCT-Ab2p) excluded from the refinement.

BRCT K1671 (Figure 3C). The phosphate group of pS406 also contributes to the dimer formation by interacting with the R1670 residue of the opposite BRCT (Figure 3C). Although a similar dimer interface was observed for BRCT-Ab1p_short structure, the cross interaction between the negative surface patch (formed by the phosphate group of pS404 and the side chain of E402) and BRCT K1671 is completely lacking.

Compared to the monomeric BRCT/Abraxas complex, where pS406 is half surrounded by BRCT1 and half exposed to the solvent, the dimerization of BRCT/Abraxas allows the second BRCT1 to reduce further the accessibility of pS406 to solvent (Figure 3E).

BRCT/2p Abraxas Complex Forms a Dimer In Vitro

To examine whether the BRCT/Abraxas complex exists as a dimer in vitro, we first tested whether dimers form in solution using size exclusion chromatography at protein concentration (1 mg/ml), much lower than the concentration used for crystallization (30 mg/ml). The elution profiles of BRCT-Ab1p and BRCT-Ab2p were different. The BRCT-Ab2p elution peak appeared to the left of the BRCT-Ab1p peak (Figure 3E), suggesting a larger hydrodynamic radius and a possible higher order BRCT-Ab2p complex. The controls show that BRCT does not interact with unphosphorylated peptide or phosphopeptide containing only phosphorylated S404 (Figure S3A). Under the same condition, the BRCT-Bach1 and BRCT-CtIP form complexes similar to that of BRCT-Ab1p (Figure 3F). Standard protein markers were also run and the positions of their elution peaks are indicated in Figure 3F. The size of BRCT-Ab2p is roughly double that of BRCT-Ab1p according to the protein markers.

We tested whether a higher concentration of BRCT-Ab1p leads to dimer formation as we observed in crystals. Indeed, the BRCT-Ab1p peak shifts left to the BRCT-Ab2p peak position once the concentration is increased (Figure S3E), indicating the tendency of BRCT-Ab1p to form higher order complexes at high protein concentrations as observed in the crystal structure. It is likely that at high concentrations, the BRCT-Ab1p complex is packed in a conformation that is not stable at low protein concentrations without a contribution from phosphorylated S404. Therefore stable dimerization of two BRCT/Abraxas complexes is unique for BRCT-Ab2p.

To confirm dimer formation, we also measured the exact molecular weight of peak fractions eluted from gel filtration using nano-electrospray mass spectrometry analysis under native conditions (Figures 3G and 3H). BRCT-Ab1p is shown to exist predominantly as a 1:1 complex with a small portion forming a 2:2 dimer. In contrast, the majority of BRCT-Ab2p is detected as 2:2 complexes, indicating a much more stable dimer. BRCT/Bach1 and BRCT/CtIP are detected only as 1:1 complexes. Consistent with the finding that higher protein concentration facilitates dimer formation (Figure S3E), the proportion of BRCT-Ab1p forming dimer increases significantly when the protein concentration is increased from 15 μM to 75 μM (Figure S3F). Small angle X-ray scattering (SAXS) experiments of the BRCT-Ab1p and BRCT-Ab2p also show similar results (Figure S4). Thus, our results indicate that only double-phosphorylated Abraxas C-terminal peptide induces stable dimerization of BRCT/Abraxas complexes in vitro.

Mutagenesis Studies of BRCT-Ab2p Dimer Interface Reveal the Importance of S404 Phosphorylation and Residues of BRCA1 Germline Mutations for Stable BRCT/Abraxas Dimer Formation

In order to test the dimer interface, we have generated various mutants for both BRCT domains and Abraxas (summarized in Figure 4C with peptide sequence indicated) based on the crystal structure. As shown in a simplified graph of the dimer interface (Figure 4A), three regions of interactions appear to contribute to formation of the dimer interface: (1) the N-terminal hydrophobic region of BRCT α1-α1; (2) extensive hydrogen bonds formed by β2-β2; and (3) the N-terminal region of Ab2p including the phosphorylated S404 interaction with BRCT α1. The interacting residues in α1-α1 and Ab2p-α1 are shown in Figure 4B.

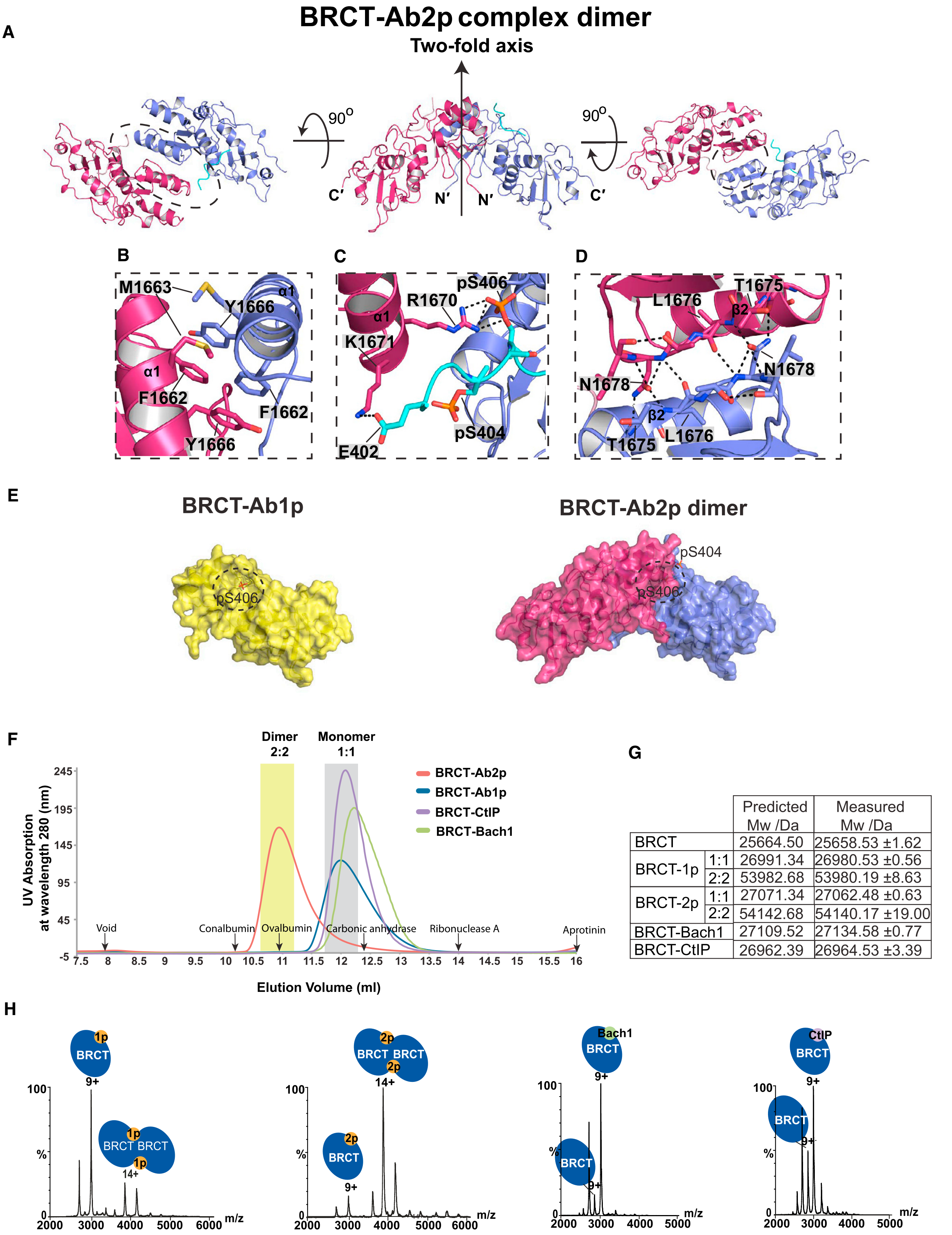

		Predicted Mw /Da	Measured Mw /Da
BRCT		25664.50	25658.53 ±1.62
BRCT-1p	1:1	26991.34	26980.53 ±0.56
	2:2	53982.68	53980.19 ±8.63
BRCT-2p	1:1	27071.34	27062.48 ±0.63
	2:2	54142.68	54140.17 ±19.00
BRCT-Bach1		27109.52	27134.58 ±0.77
BRCT-CtIP		26962.39	26964.53 ±3.39

(legend on next page)

By size exclusion chromatography, we first tested the importance of S404 phosphorylation. Mutations of Abraxas S404 to proline or aspartic acid were generated. While BRCT-Ab1p (S404P) leads to 1:1 complex formation, BRCT-Ab1p (S404D) can maintain the 2:2 complex dimer as BRCT-Ab2p (Figure 4D). This confirms that S404 phosphorylation is essential for dimerization. A previous report using optimized peptide containing aspartic acid in the equivalent position to Abraxas S404 was reported to not form a dimer in solution (Williams et al., 2004). We reasoned that the difference between the optimized peptide and Ab2p is in the N-terminal region ($G^{399}F^{400}G^{401}E^{402}$) of Ab2p. We demonstrated that BRCT-Ab2p_short without GFGE still forms a dimer indicating that GEGE is not absolutely required for dimerization when Y^{403} and pS^{404} are present. However, when we analyzed BRCT-Ab2p in solution, we found that while F400D did not affect dimer formation, E402R, as well as BRCT K1671E or R1670E, partially destabilizes dimer formation. Double mutation of BRCT(K1671E)-Ab2p(E402R) further destabilized the complex, leading to elution at a position close to that of 1:1 stoichiometry (Figure 4E). These results indicate that although GFGE is not absolutely required for the dimer formation, it contributes to the dimer stabilization when it is present.

We also tested the importance of the BRCT P1659 interaction with Abraxas Y403 to the stability of the BRCT-Ab2p dimer. In the presence of the N-terminal region (GFGE) of Ab2p, mutation of Abraxas Y403A did not destabilize the dimer complex formation in either BRCT-Ab2p(Y403A) or BRCT(P1659G)-Ab2p(Y403A). But when the N-terminal region of Abraxas was absent, the dimer complex of BRCT-Ab2p(Y403A_short) became unstable. This destabilization was further enhanced in BRCT(P1659G)-Abraxas(Y403A)_short complex, which was eluted near the 1:1 complex (Figure 4F). Together, these results are consistent with the crystal structure analysis showing that Ab2p promotes dimer formation in two different ways: (1) phosphorylated S404 fixes the side chain of Y403, which generates additional interaction with BRCT K1671 and (2) the phosphorylated group of pS404 and E402 form a negative surface region that leads to cross interaction with BRCT K1671.

We then evaluated the contribution of the hydrophobic interactions between the α helices (α1-α1) and the extensive hydrogen bonds between the two antiparallel β strands (β2-β2) in the two protomers that comprise the dimer (Figure 4G). Our results indicate that the α1-α1 interaction contributes more significantly toward the stabilization of the dimer interface than the β2-β2 interaction. The BRCT mutant N1678A, which disrupted the β2-β2 interaction (Figure 3D), reduced hydrogen bonds between the side chain of N1678 and the nearby residue T1675, but did not destabilize BRCT-Ab2p dimer formation. In contrast, F1662S and M1663K, two BRCA1 germline mutations identified in cancer patients (Szabo et al., 2000), led to complete disruption of the dimer formation as the elution peaks of these two mutants in complex with Ab2p moved to the 1:1 complex position. BRCT Y1666A mutant did not result in complete disruption of the dimer and the peak in the elution profile is located between that for the 2:2 and 1:1 complexes. These results support our observation from the crystal structure that F1662S and M1663K have a much more significant effect on disruption of the dimer interface because these two residues are located at the point of the isologous dimer interface, while Y1666A is further away.

Abraxas S404 Is Important for Cellular Resistance to IR and Accumulation of BRCA1 at the DNA Damage Site

Since IR-induced phosphorylation of S404 appears to promote stable BRCT/Abraxas dimer formation, the S404 residue is likely to be critical for the function of Abraxas in response to IR. We first tested whether S404 is important for the cellular response to IR. In an IR sensitivity assay, both S404A and S406A mutants of Abraxas were unable to fully rescue the increased sensitivity of Abraxas knockdown cells as the wild-type Abraxas did (Figures 5A 5B, and S5), suggesting that phosphorylation of S404 plays a role in the cellular resistance to IR. Abraxas recruits BRCA1 to DNA damage sites in response to IR. We thus examined the role of S404 phosphorylation in BRCA1 accumulation at the DNA damage sites. The percentage of cells containing more than ten BRCA1 IR-induced foci (IRIF), as well as the intensity of the foci, were significantly decreased in Abraxas knockdown cells. While the defect of Abraxas knockdown cells in BRCA1 IRIF formation could be rescued by expression of wild-type Abraxas, it could not be rescued by expression of Abraxas S406A or S404A mutant (Figures 5C–5E and S5). Consistently, when we examined the chromatin-bound BRCA1 levels in response to IR, we found that both the S404A and S406A mutants failed to accumulate BRCA1 to damaged chromatin as the wild-type Abraxas does (Figure 5F). As a control, the total expression level of BRCA1 was not affected in Abraxas knockdown cells or the cells complemented with expression of either wild-type or mutant Abraxas. Thus, phosphorylation of S404 is likely to play an important role in BRCA1 accumulation to DNA damage sites and in cellular resistance to IR.

Figure 3. 2p Ab Induces Dimerization of BRCT-Ab2p Complex

(A) Crystal structure of BRCT-Ab2p complex dimer viewed from three different directions with a two-fold axis. The dimer interface is within the dashed circles and zoomed in (B)–(D).

(B) Dimer interface between two BRCT α1 helices.

(C) Interaction between BRCT α1 helix and Ab2p.

(D) Dimer interface between two BRCT β2 strands. The polar interactions between labeled residues are shown in black dashed lines. The key residues are indicated in the image.

(E) Surface representation of BRCA1-1p_short (yellow) and BRCT-Ab2p dimer (blue and pink). The Abraxas pS406 binding region is indicated in the dashed circle.

(F) Gel filtration BRCT in complex with Ab1p, Ab2p, Bach1, and CtIP phosphopeptides at a concentration of 40 μM (1 mg/ml). The regions for dimer complex (2:2 complex) and monomer complex (1:1 complex) are high lined in yellow and gray shades. The elution positions for void and protein markers aprotinin (Mw = 6,500 Da), ribonuclease A (Mw = 13,700 Da), carbonic anhydrase (Mw = 29,000 Da), Ovalbumin (Mw = 44,000 Da), and Conalbumin (Mw = 75,000 Da) are indicated.

(G) The molecular weight of BRCT and its complexes with phosphopeptide, measured using native mass spectrometry.

(H) The native mass spectra of BRCT-Ab1p, BRCT-Ab2p, BRCT-Bach1, and BRCT-CtIP complexes tested at 15 μM (see also Figures S3 and S4).

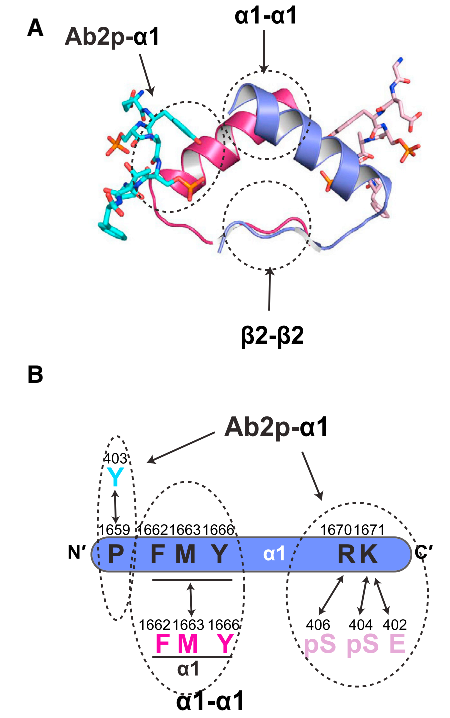

C

	BRCT	Abraxas	
Ab2p-α1	BRCT	Ab2p_short	YpSRpSPTF
	BRCT	Ab1p(S404P)	GFGEYPRpSPTF
	BRCT	Ab1p(S404D)	GFGEYDRpSPTF
	BRCT	Ab2p(F400D)	GDGEYpSRpSPTF
	BRCT	Ab2p(E402R)	GFGRYpSRpSPTF
	R1670E	Ab2p	GFGEYpSRpSPTF
	K1671E	Ab2p	GFGEYpSRpSPTF
	K1671E	Ab2p(E402R)	GFGRYpSRpSPTF
	BRCT	Ab2p(Y403A)	GFGEApSRpSPTF
	BRCT	Ab2p(Y403A)short	ApSRpSPTF
	P1659G	Ab2p(Y403A)	GFGEApSRpSPTF
	P1659G	Ab2p(Y403A)short	ApSRpSPTF
α1-α1	F1662S	Ab2p	GFGEYpSRpSPTF
	M1663K	Ab2p	GFGEYpSRpSPTF
	Y1666A	Ab2p	GFGEYpSRpSPTF
β2-β2	N1678A	Ab2p	GFGEYpSRpSPTF

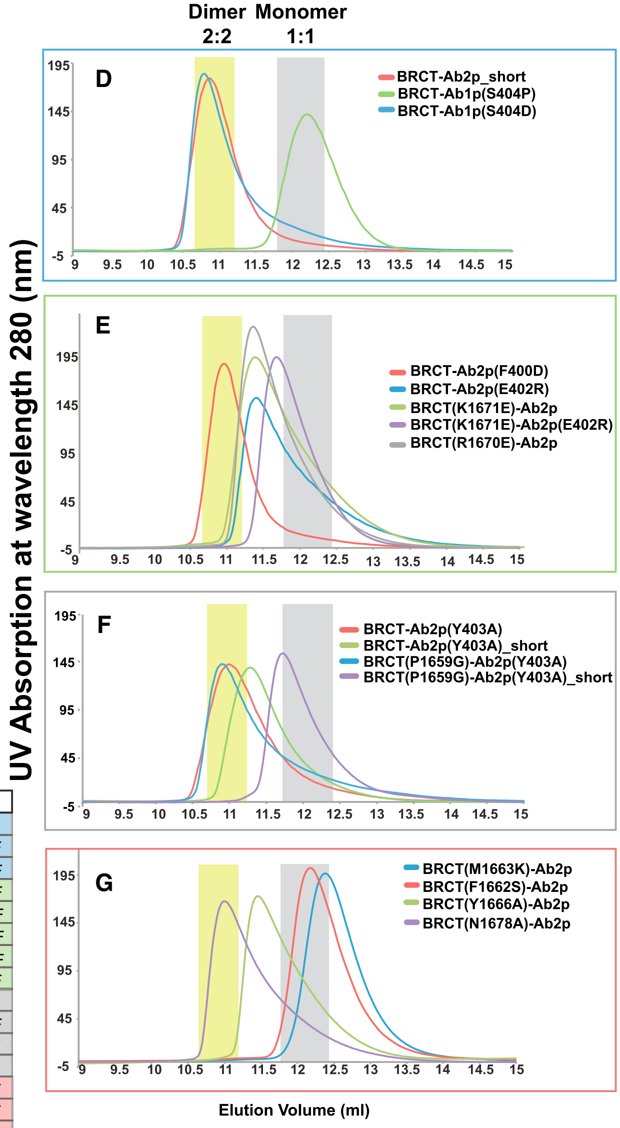

Figure 4. Mutagenesis Studies of the Interface of BRCT-Ab2p Complex Dimer

(A) Simplified BRCT-Ab2p dimer interface containing three regions observed in BRCT-Ab2p crystal.

(B) Detailed interactions mediated through BRCT $\alpha1$.

(C) Summary of BRCT and Abraxas mutants. The complexes tested are grouped into four and highlighted in different colors.

(D–G) Gel filtrations of BRCT and Abraxas mutants. The same color codes are used as in (C).

Abraxas-Dependent BRCA1 Dimerization In Vivo

We tested whether BRCA1 forms dimers in vivo and whether the stable dimer formation is dependent on Abraxas. We co-expressed differentially Myc- or FLAG-tagged BRCA1 full-length protein in control (Ctrl) cells or Abraxas knockout (KO) cells. In the co-immunoprecipitation experiment with lysates prepared from cells treated with IR, immunoprecipitated FLAG-BRCA1 interacts with Myc-tagged BRCA1, indicating that BRCA1 indeed dimerizes in vivo. The dimerization was decreased in Abraxas KO cells, indicating the dependency of dimerization on Abraxas (Figure 6A). Similarly, a construct containing only the BRCA1-BRCT domains also dimerizes when co-expressed in cells and the dimerization is decreased in Abraxas KO cells (Figure 6B).

We then tested whether the germline mutations F1662S and M1663K interfere with BRCA1 dimerization in vivo. We compared the interaction of a Myc-tagged full-length BRCA1 and a HA-tagged wild-type BRCT fragment with that of the F1662S or M1663K mutant of BRCA1 and a mutant BRCA1 BRCT fragment with three residues localized in the dimer interface mutated (F1662S/M1663K/R1670E). Both the Myc-immunoprecipitation (Figure 6C) and reciprocal HA-immunoprecipitation (Figure 6D) experiments showed that the

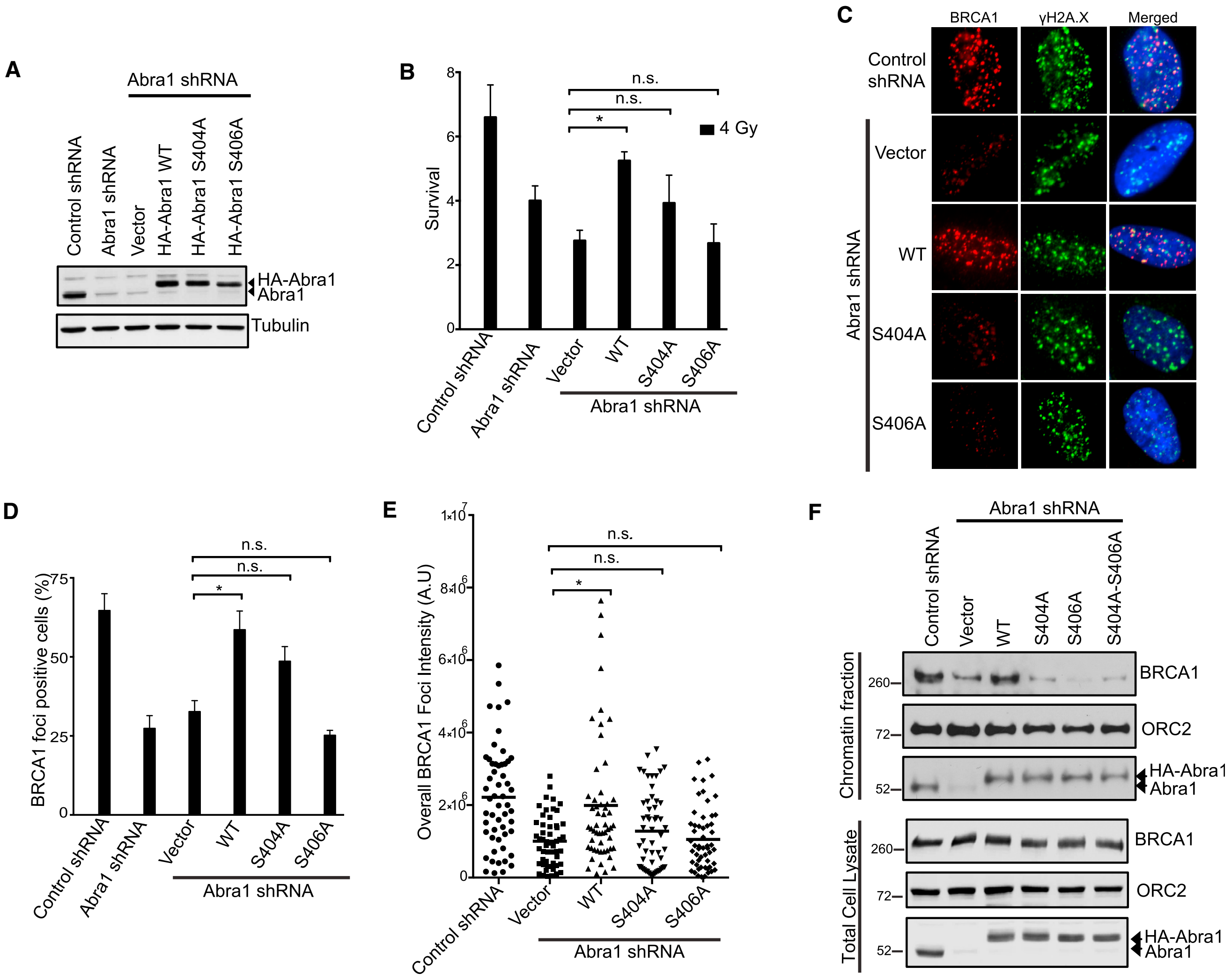

Figure 5. Phosphorylation of S404 and S406 Are Both Important for Cellular Resistance to IR and BRCA1 Accumulation at DNA Damage Sites

(A) Generation of Abraxas knockdown U2OS cells complemented with expression of small hairpin (sh)RNA-resistant HA-tagged WT, S404A, or S406A mutants of Abraxas.

(B) Increased cellular sensitivity to IR of Abraxas-deficient cells expressing mutants of Abraxas. The colony-survival assay was carried out for cells treated with 4 Gy IR. The data are presented as means ± SD. The data analyses are processed by ANOVA and the statistical significance was determined by Tukey's multiple comparisons test (*p < 0.02). There were three independent experiments that were performed (additional data are presented in Figure S5).

(C) Representative images of BRCA1 IRIF in Abra1 shRNA knockdown cells complemented with vector, WT, or mutants of Abraxas in response to 10 Gy IR followed by 2 hr incubation at 37°C.

(D) The percentage of cells containing more than ten BRCA1 IRIF foci was quantified. The data are presented as means ± SD. The data analyses are processed by ANOVA and the statistical significance was determined by Tukey's multiple comparisons test (*p < 0.0001). At least three independent experiments were performed. More than 300 cells were counted for each experiment. Additional data for quantification at different time points post IR are presented in Figure S5.

(E) Quantification of the intensity of BRCA1 IR induced foci (IRIF). The data are presented as means ± SD (n > 50). The statistical analysis was carried out by Student's t test (*p < 0.0002).

(F) BRCA1 accumulation at damaged chromatin depends on both S404 and S406 residues. The Orc2 was used as a marker for chromatin-bound fraction. The cells were treated with 10 Gy IR followed by 2 hr incubation at 37°C. The cellular fractionation was carried out and the chromatin fraction was analyzed (see also Figure S5).

interaction/dimerization of BRCA1 and BRCT was decreased with mutation of the critical residues at the dimer interface F1662S or M1663K. Thus, BRCA1 germline mutations interfere with stable dimer formation in vivo.

To understand dimerization of the BRCT/Abraxas complex in vivo, we examined whether Abraxas forms a dimer in which the phosphorylated C-termini of Abraxas in complex with BRCT could be in close vicinity for dimerization. We expressed both GFP-tagged Abraxas and HA-FLAG-tagged Abraxas in cells and tested whether the differentially tagged Abraxas molecules interact with each other using the immunoprecipitation assay. We found that wild-type Abraxas, as well as the S404A

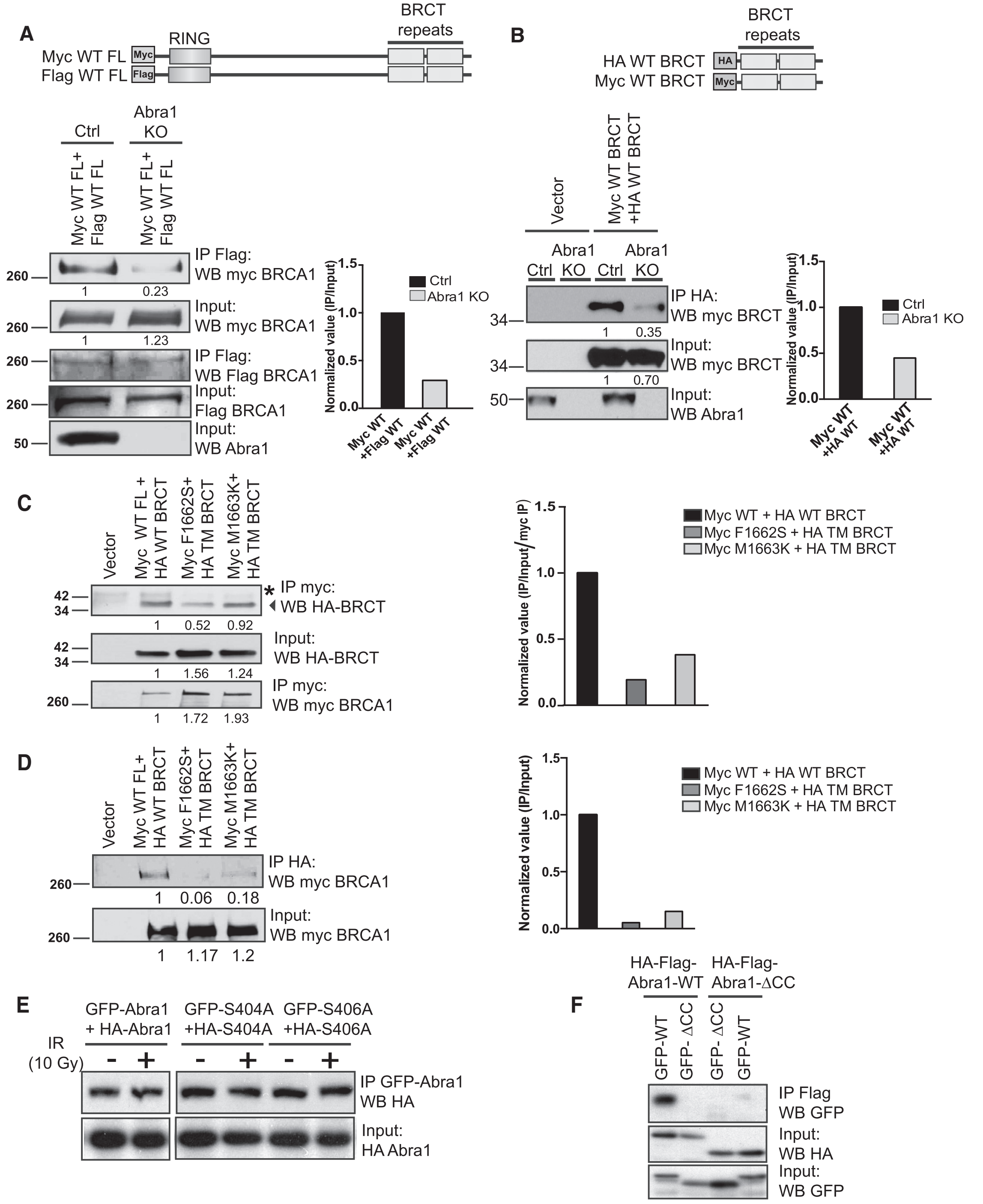

Figure 6. Abraxas Promotes BRCA1 BRCT Dimerization In Vivo

(A) Abraxas-dependent BRCA1 dimerization in vivo. The differentially Myc- and FLAG-tagged BRCA1 full-length constructs were transiently transfected into parental 293T (Ctrl) or Abraxas KO cells. The lysates from cells treated with 10 Gy IR followed by 1 hr incubation at 37°C were used for FLAG-immunoprecipitation. The intensity of individual bands was quantified by densitometric analysis using NIH ImageJ software. The normalized value (IPed_mycBRCA1/Input_mycBRCA1) was shown in the bar graph.

(B) Abraxas-dependent BRCA1-BRCT domains dimerization in vivo. The differentially Myc- and HA-tagged BRCA1-BRCT domains constructs were transiently transfected into parental 293T (Ctrl) or Abraxas KO cells. The lysates from cells treated with 10 Gy IR followed by 1 hr incubation at 37°C were used for HA-immunoprecipitation. The band intensity was quantified with NIH imageJ software. The normalized value (IPed_mycBRCT/Input_mycBRCT) was shown in the bar graph.

(legend continued on next page)

and S406A mutant, interact with differentially tagged counterpart, indicating that Abraxas dimerizes/oligomerizes in vivo independently of its binding to BRCA1 (Figure 6E). We then investigated what region of Abraxas mediates the dimerization by examining various deletion mutants of Abraxas. Deletion of the CC domain abolished the self-interaction of either wild-type or mutant Abraxas (Figures 6F and S6). Thus, in vivo, Abraxas dimerizes/oligomerizes through the CC domain.

DISCUSSION

BRCA1 accumulation to DNA damage sites is a crucial step for BRCA1's function in DNA damage repair, and BRCT domains of BRCA1 are important for the tumor suppressor function of BRCA1. IR-induced ubiquitination at DNA damages sites generates docking sites for the recruitment of the Abraxas/BRCA1-A complex and accumulation of BRCA1 at sites of damage. Our data provide evidence for an IR-induced, ATM-dependent mechanism specific to Abraxas-mediated recruitment of BRCA1. In such a model (Figure 7), IR-induced phosphorylation of S404 next to the pSPxF induces stable dimer formation of the BRCA1 BRCT/Abraxas complex.

The crystal structural analysis of BRCT in complex with Abraxas phosphorylated peptides revealed that, although both Ab1p and Ab2p bind to BRCT domains through the same pSPxF motif, the phosphorylation of S404 in Ab2p induces stable dimerization of the BRCT/Abraxas complex. The dimer interface locates to the BRCT1 of BRCA1 tandem BRCT domains. As expected from previous reports, BRCT1 also provides the interaction site for pS406 of the pSPxF motif, while the side chain of F of the motif inserts into a hydrophobic pocket created mainly by BRCT2 domain. The dimer surface formed between two BRCT1 domains does not directly influence the interaction between the pSPxF motif and the BRCT domains. Although the hydrophobic α1 interface observed in the BRCT-Ab2p dimer was buried in a similar manner to that of the BRCT-Ab1p or other BRCA1 BRCT related crystal structures (Wu et al., 2015), this interface alone is not strong enough to form a stable dimer in solution as we observed for BRCT domain only, BRCT-Ab1p, BRCT-Bach1, or BRCT-CtIP. In contrast, under the same condition, the stable-dimer state of BRCT-Ab2p is triggered by the phosphorylation of S404. A phosphorylation-mimetic point mutant S404D stabilizes BRCT/Abraxas dimer formation in solution in a similar way to phosphorylated S404, further supporting the conclusion that phosphorylation of S404 promotes dimer formation. The impact of phosphorylation of Abraxas S404 is the following: (1) the highly charged phosphate group of pS404 faces away from BRCT domains, resulting in stabilization of the interaction of the side chain of Y403 with BRCT P1659 located at the N terminus of α1. This interaction causes the shift of α1 closer to the N terminus of the phosphopeptide; and (2) the negatively charged side chains of pS404 and E402 provide extra ionic interaction sites with K1671 of BRCT. All together, this leads to a stable BRCT-Ab2p complex dimer formation. Owing to the symmetric pairing shape, we describe the BRCT-Ab2p dimerization interaction as a "pair-hugging" interaction mode, in which the Abraxas phosphopeptide acts as an arm wrapping around the other BRCT domain therefore stabilizing the interaction (Figure 7).

The IR-induced phosphorylation of Abraxas S404 and the subsequent stable BRCA1-BRCT dimerization are likely to comprise an important mechanism for cellular response to IR since mutation of S404 leads to decreased BRCA1 accumulation to DNA damaged chromatin and increased cellular sensitivity to IR. IR-induced phosphorylation of Abraxas S404 may facilitate the accumulation of BRCA1 at DNA damage sites by stabilizing the BRCA1 protein dimerization, forming more stable higher order complexes at sites of damage. In addition, S404 phosphorylation may further facilitate the interaction of Abraxas and BRCA1 by reducing the dissociation of pS406, so prolonging the Abraxas interaction with BRCT domains or limiting the accessibility of pS406 by other proteins such as phosphatase. Alternatively, induced dimerization of BRCA1 BRCT by phosphorylation of S404 of Abraxas could increase the local concentration of BRCA1 at damaged chromatin, which is likely critical for efficient DNA damage signaling and repair.

Many tumor-derived truncation and missense mutations have been identified in the BRCA1 BRCT domains. While some of these mutations have been shown to either destabilize the protein fold of the BRCT domains or disrupt the binding surface to pSPxF-containing phosphopeptides (Cantor et al., 2001; Clapperton et al., 2004; Coquelle et al., 2011; Manke et al., 2003; Shiozaki et al., 2004; Williams and Glover, 2003; Williams et al., 2004; Yu et al., 2003), resulting in cancer predisposition, the function of a large number of BRCT mutations is still unknown (Easton et al., 2007). Our analyses reveal that germline mutations F1662S and M1663K disrupt the ability of BRCT to dimerize, in vitro and in vivo, providing a structural explanation for the possible role of these mutations in inactivating BRCA1 tumor suppressor function. Future study is needed to further characterize the effect of these mutations in the function of BRCA1 in DNA repair and damage signaling.

How is the dimerization of BRCT-Abraxas achieved in vivo? Since the phosphorylation of S406 is not IR-dependent, Abraxas binds to BRCA1 through the pSPxF motif even in the absence of DNA damage (Wang et al., 2007). The dimerization of Abraxas

(C and D) BRCA1 germline mutations F1662S or M1663K decrease BRCA1 dimerization in vivo. Myc-tagged BRCA1 full-length (WT-FL) and HA-tagged BRCA1 BRCT (WT-BRCT) or Myc-tagged mutant full-length (F1662S or M1663K) and HA-tagged BRCT triple mutant (TM, F1662S/M1663K/R1670E) were co-expressed in cells. The lysates from cells treated with 10 Gy IR followed by 1 hr incubation at 37°C were prepared for either Myc- immunoprecipitation (C) or reciprocal IP with HA- immunoprecipitation (D). The normalized value was shown in the bar graph.

(E) Abraxas dimerize/oligomerize in vivo independent of binding to BRCA1. The differentially tagged GFP- and HA-FLAG-tagged WT or GFP-tagged and HA-FLAG-tagged Abra1 mutants (S404A or S406A) were co-expressed in cells. The anti-GFP immunoprecipitation was carried out with lysates from 293T cells treated or not treated with 10 Gy IR followed by 1 hr incubation at 37°C. The images are from the same blot.

(F) Abraxas dimerizes/oligomerizes, in vivo, through the CC domain. The immunoprecipitations were carried out with lysates prepared from cells co-expressing HA-FLAG-tagged WT Abra1 or HA-FLAG-tagged CC domain deletion mutant (ΔCC) and GFP-tagged WT or ΔCC (see also Figure S6).

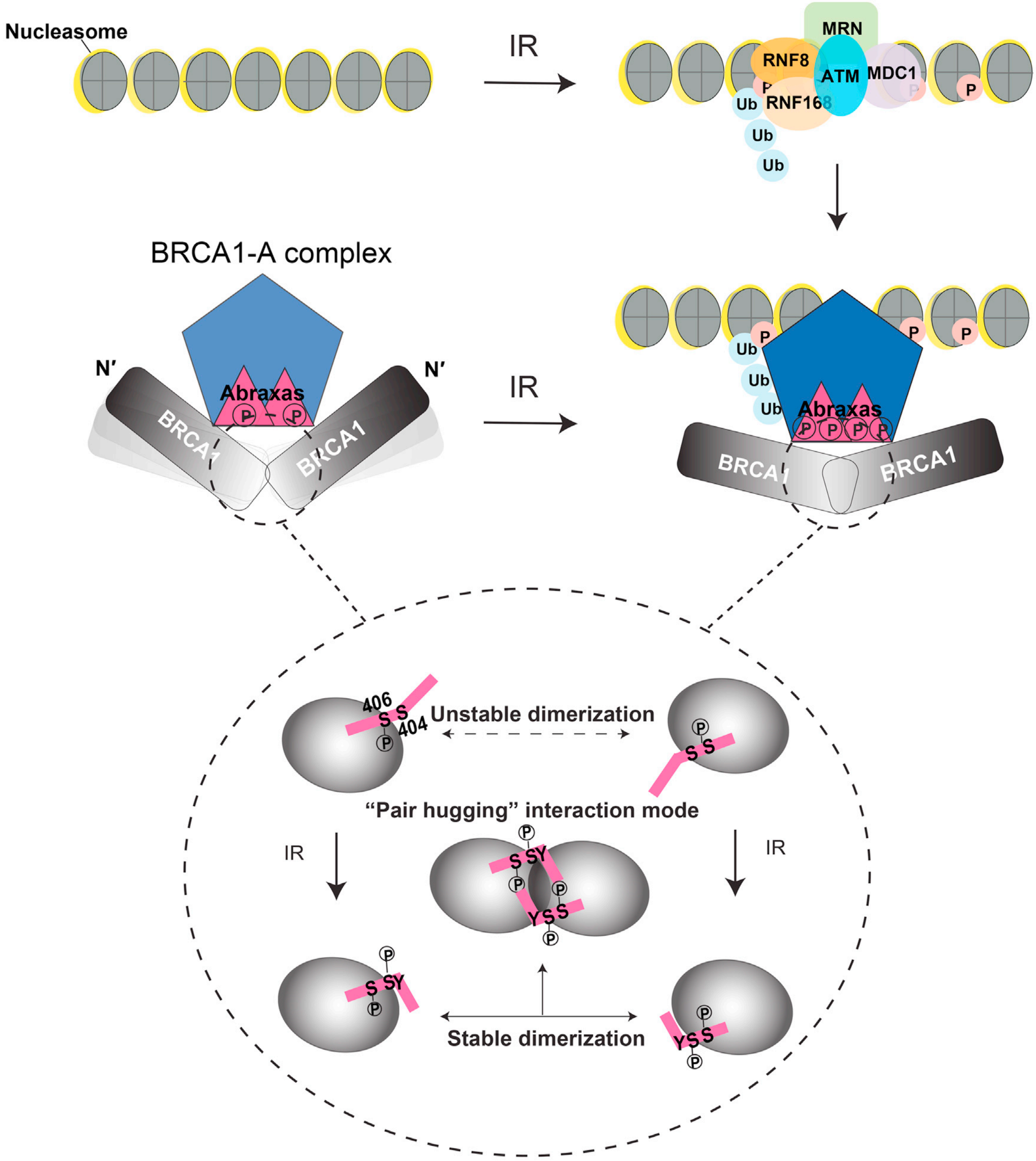

Figure 7. A Model Showing IR-Induced Phosphorylation of Abraxas Promotes Dimerization of BRCA1 at Sites of DNA Damage for BRCA1 Accumulation and Cellular Response to IR

In the absence of IR, two BRCA1 bound to BRCA1-A complex do not form stable dimer. Extra phosphorylation at Abraxas S404 induced by IR leads to the stable dimerization of BRCA1 at the DNA damaged site. The BRCT-Abraxas interaction is indicated in a dashed circle for the "pair-hugging" interaction model. The BRCT domains are represented by gray circles and Abraxas phosphopeptides by pink lines. The line with double arrows indicates interaction for dimerization (P indicates the phosphorylation).

through the CC domain could potentially position the two BRCT tandem domains that interact at the C terminus of Abraxas into close vicinity, leading to an unstable dimer of the BRCT-Abraxas complex in the absence of DNA damage. In response to IR, IR-induced phosphorylation of S404 leads to an increase of affinity between the phosphate group and the residues at the dimer surface, which consequently results in a much more stable dimer complex of BRCA1 BRCT/Abraxas. Since the CC domain of Abraxas also appears to dimerize with the CC domain of BRCC36 (Wang and Elledge, 2007), it is likely that, in the BRCA1-A complex, Abraxas and BRCC36 form an oligomeric bundle through the CC domain present on each of the Abraxas and BRCC36 molecules. Future structural analysis of the BRCA1-A complex is needed to further understand the

multimerization of Abraxas and BRCC36 of the BRCA1-A complex. Nevertheless, IR-induced phosphorylation at Abraxas S404 appears to function as a regulatory switch, which leads to stable dimerization of two nearby BRCT domains.

The phosphorylation-induced BRCT dimerization is observed only in the BRCT/Abraxas complex. We demonstrate that, in addition to the pSPxF-binding motif, IR-induced phosphorylation of a nearby S404 residue can further regulate the interaction of BRCT and Abraxas. Thus, amino acid sequences outside the pSPxF motif may confer specificity in regulation of the BRCT binding to phosphorylated proteins. Indeed, in addition to phosphorylated S404, the N-terminal region of Ab (GFGE^{402}Y^{403}pS^{404}RpSPVF) also contributes to stable BRCT/Abraxas dimer formation. The E402 residue cooperates with the phosphorylated S404 in stabilizing the dimer formation; the side chain of Y403 is fixed to interact with BRCT P1659 when S404 is phosphorylated. Thus, the unique sequence feature outside of the pSPxF motif ensures that stable dimer formation occurs only with BRCT/Abraxas, but not with other BRCT complexes. Since Abraxas-BRCA1 interaction has been shown critical for DNA repair and maintenance of genomic stability, the additional regulatory mechanism uncovered in this study in regulating the interaction of Abraxas and BRCA1 further highlights the importance of this interaction in BRCA1 signaling and tumor suppression.

In summary, our study reveals a phosphorylation-dependent mechanism in Abraxas-mediated recruitment and accumulation of BRCA1 at DNA damage sites, deepening our understanding of BRCA1 and Abraxas tumor suppressor function and related cellular signaling. Our study also provides structural insights that will assist the design of small molecules modulating BRCA1-Abraxas interaction in the future.

EXPERIMENTAL PROCEDURES

Cell Lines, Culture, and Antibodies

U2OS cells were cultured in McCoy's 5A medium supplemented with 10% fetal bovine serum (FBS). 293T cells were grown in Dulbecco's modified Eagles medium (DMEM) supplemented with 10% FBS. Details of generation of Abraxas knockdown or KO cells are described in the Supplemental Information. Antibodies used are described in the Supplemental Information.

Immunofluorescence

Cells were fixed with 3.6% formaldehyde for 10 min, permeabilized with 0.5% Triton X-100 solution, and incubated with primary antibodies for 1 hr at 37°C followed by appropriate Alexa 488-conjugated (green; Invitrogen) and Alexa 555-conjugated (red; Invitrogen) secondary antibodies. Additional information is included in the Supplemental Information.

Cell Lysis and Immunoprecipitation

Cell lysates were prepared from 293T cells or 293T Abra1-gene KO cells either untreated or treated with 10 Gy IR followed by incubation at 37°C for 1 hr. FLAG immunoprecipitation (IP) was performed with lysates prepared from cells either treated or untreated with 10 Gy IR followed by 1 hr incubation at 37°C. Western blots were carried out using indicated antibodies. 293T cells were incubated with kinase inhibitors for 2 hr at indicated concentrations. Cells were then either exposed to 4 Gy IR or untreated. After 1 hr incubation, cells were lysed and Abraxas pS404pS406 levels were determined by western blot. Additional information is included in the Supplemental Information.

Clonogenic Survival Assay

Stable U2OS cell lines were seeded at low density in 10 cm dishes and irradiated with 4 Gy ionizing irradiation using a ^{137}Cs source. The cells were then cultured at 37°C for 14 days to allow colonies to form. Colonies were stained with 2% methylene blue and 50% ethanol for 10 min.

Chromatin Fractionation

Abraxas knockdown cells complemented with vector, wild-type (WT), S404A, S406A, and double mutant (S404A and S406A) were irradiated at 10 Gy and incubated for 1 hr at 37°C. Cells were then subjected for chromatin fractionation followed by detection with indicated antibodies. Details are described in the Supplemental Information.

BRCT Construct, Purification, Crystallization, and Data Collection

Construction and purification of BRCA1 BRCT and mutants are described in the Supplemental Information. Purified BRCT was mixed with Abs at a 1:3 molar ratio as has been previously reported (Shiozaki et al., 2004) and incubated at 4°C for 30 min. Final protein concentration was 30 mg/ml. Crystallization was set up using the hanging-drop vapor diffusion method with drops containing 1 μl of protein sample and 1 μl of crystallization solution. Crystals appeared after 3–4 days. BRCT-Ab2p was crystallized in 0.1 M HEPES (pH 7.0), 60 mM ammonium sulfate, and 5% (w/v) PEG 4000. BRCT-Ab1p_short was crystallized in 1 M lithium chloride, 0.1 Tris (pH 8.0), and 20% (w/v) PEG 6000. X-ray diffraction data were processed by XDS (Kabsch, 2010) and Scala (Winn et al., 2011). The phases for the structure factors were obtained through molecular replacement using Phaser module in Phenix 1.8.4-1496 (Adams et al., 2010). More detail of protein crystal and structure determination can be found in Supplemental Information.

Size Exclusion Chromatography for BRCT with Abraxas, Bach1, and CtIP Phosphopeptides

BRCT protein and Abraxas, Bach1, and CtIP peptides were mixed in a 1:3 molar ratio to a final concentration of 1 mg/ml (about 40 μM) in 500 μl loops. Gel filtration was performed in Buffer A using Superdex 75 10/300 column (GE Healthcare life) with a flow rate of 0.5 ml/min. For studying the high protein concentration effects on BRCT-Abraxas complex formation, a final concentration of 10 mg/ml was used. Protein markers (GE Gel Filtration LMW Calibration Kit) were run following the kit protocol.

Native Mass Spectrometry

Samples were diluted to 15 or 75 μM protein concentration in 300 mM ammonium acetate (pH 7.6) and further buffer exchanged into 300 mM ammonium acetate using Bio-Spin 6 (Bio-Rad) column. The desalted samples were loaded into the in-house prepared gold-coated glass capillaries (Hernández and Robinson, 2007). Nano-electrospray mass spectrometric analyses were performed under native conditions on a hybrid quadrupole time-of-flight mass spectrometer previously modified for high mass transmission (Sobott et al., 2002). Typically the following instrumental conditions were used: capillary voltage 1.3 kV, sample cone 200 V, and collision cell energy 5 V.

ACCESSION NUMBERS

The accession numbers for the BRCT-Ab1p_short and BRCT-Ab2p data reported in this paper are PDB: 4Y2G, 4Y18.

SUPPLEMENTAL INFORMATION

Supplemental Information includes Supplemental Experimental Procedures and six figures and can be found with this article online at http://dx.doi.org/10.1016/j.molcel.2015.12.017.

AUTHOR CONTRIBUTIONS

Q.W., B.W., and T.L.B. initiated this project. Q.W. performed most of the in vitro work and solved structures. A.P., D.S., and B.W. designed the in vivo experiments. D.S. generated Abraxas KO cells and analyzed Abraxas double

phosphorylation in response to IR and in the presence of kinase inhibitors, as well as Abraxas dimerization in vivo. A.P. generated Abraxas knockdown cells, examined the Abraxas mutants in rescuing the defects of Abraxas-deficient cells, and Abraxas-dependent BRCA1 dimerization in vivo. S.M. and C.V.R. performed and analyzed the native mass spectrometry experiment. T.O. conducted SAXS experiment. E.L.B. made and purified the BRCT P1659G mutant. T.K.F. and B.X. generated constructs expressing Myc-tagged BRCA1 F1662S and M1663K mutants. Q.W., A.P., B.W., and T.L.B. wrote the paper together with comments from other co-authors.

ACKNOWLEDGMENTS

We thank beamline scientists at Diamond Light Source for help during data collection of crystals and SAXS. The crystallization experiments were performed in the Crystallographic X-ray Facility at the Department of Biochemistry, University of Cambridge. We are grateful for the advice and help from Facility Manager Dr. Dimitri Chirgadze. We also thank Dr. Yanfen Hu (University of Texas Health Science Center at San Antonio) for the pFLAG-BRCA1 plasmid and Dr. Angela Pacitto (University of Cambridge) for reading the manuscript. We thank Dr. Adriana Paulucci-Holthanuzen (Department of Genetics-MD Anderson Microscopy Core Facility) for assistance with images and analysis. Q.W., T.O., and T.L.B. are funded by the Wellcome Trust (grant 093167/Z/10/Z). A.P. is an awardee of the Schissler Foundation Fellowship, the Center for Cancer Epigenetics Scholarship, and the Andrew Sowell-Wade Huggins Scholarship. This work is supported by the NIH grant (CA155025 to B.W.) with funds from the University of Texas MD Anderson Cancer Center (IRG, Center for Cancer Epigenetics, Center for Genetics, and Genomics Pilot Award). S.M. is funded by the Medical Research Council (grant 98101 to C.V.R.) and C.V.R. is a Royal Society Research Professor. T.K.F. and B.X. are supported by NIH grant (R01CA138804 to B.X.).

Received: June 12, 2015
Revised: October 22, 2015
Accepted: December 9, 2015
Published: January 14, 2016

REFERENCES

Adams, P.D., Afonine, P.V., Bunkóczi, G., Chen, V.B., Davis, I.W., Echols, N., Headd, J.J., Hung, L.W., Kapral, G.J., Grosse-Kunstleve, R.W.G., et al. (2010). PHENIX: a comprehensive Python-based system for macromolecular structure solution. Acta Crystallogr. D Biol. Crystallogr. *66*, 213–221.

Campbell, S.J., Edwards, R.A., and Glover, J.N.M. (2010). Comparison of the structures and peptide binding specificities of the BRCT domains of MDC1 and BRCA1. Structure *18*, 167–176.

Cantor, S.B., Bell, D.W., Ganesan, S., Kass, E.M., Drapkin, R., Grossman, S., Wahrer, D.C.R., Sgroi, D.C., Lane, W.S., Haber, D.A., and Livingston, D.M. (2001). BACH1, a novel helicase-like protein, interacts directly with BRCA1 and contributes to its DNA repair function. Cell *105*, 149–160.

Castillo, A., Paul, A., Sun, B., Huang, T.H., Wang, Y., Yazinski, S.A., Tyler, J., Li, L., You, M.J., Zou, L., et al. (2014). The BRCA1-interacting protein Abraxas is required for genomic stability and tumor suppression. Cell Rep. *8*, 807–817.

Clapperton, J.A., Manke, I.A., Lowery, D.M., Ho, T., Haire, L.F., Yaffe, M.B., and Smerdon, S.J. (2004). Structure and mechanism of BRCA1 BRCT domain recognition of phosphorylated BACH1 with implications for cancer. Nat. Struct. Mol. Biol. *11*, 512–518.

Coquelle, N., Green, R., and Glover, J.N.M. (2011). Impact of BRCA1 BRCT domain missense substitutions on phosphopeptide recognition. Biochemistry *50*, 4579–4589.

Couch, F.J., and Weber, B.L. (1996). Mutations and polymorphisms in the familial early-onset breast cancer (BRCA1) gene. Breast Cancer Information Core. Hum. Mutat. *8*, 8–18.

Doil, C., Mailand, N., Bekker-Jensen, S., Menard, P., Larsen, D.H., Pepperkok, R., Ellenberg, J., Panier, S., Durocher, D., Bartek, J., et al. (2009). RNF168 binds and amplifies ubiquitin conjugates on damaged chromosomes to allow accumulation of repair proteins. Cell *136*, 435–446.

Easton, D.F., Deffenbaugh, A.M., Pruss, D., Frye, C., Wenstrup, R.J., Allen-Brady, K., Tavtigian, S.V., Monteiro, A.N., Iversen, E.S., Couch, F.J., and Goldgar, D.E. (2007). A systematic genetic assessment of 1,433 sequence variants of unknown clinical significance in the BRCA1 and BRCA2 breast cancer-predisposition genes. Am. J. Hum. Genet. *81*, 873–883.

Friedman, L.S., Ostermeyer, E.A., Szabo, C.I., Dowd, P., Lynch, E.D., Rowell, S.E., and King, M.-C. (1994). Confirmation of BRCA1 by analysis of germline mutations linked to breast and ovarian cancer in ten families. Nat. Genet. *8*, 399–404.

Futreal, P.A., Liu, Q., Shattuck-Eidens, D., Cochran, C., Harshman, K., Tavtigian, S., Bennett, L.M., Haugen-Strano, A., Swensen, J., Miki, Y., et al. (1994). BRCA1 mutations in primary breast and ovarian carcinomas. Science *266*, 120–122.

Glover, J.N.M., Williams, R.S., and Lee, M.S. (2004). Interactions between BRCT repeats and phosphoproteins: tangled up in two. Trends Biochem. Sci. *29*, 579–585.

Hall, J.M., Lee, M.K., Newman, B., Morrow, J.E., Anderson, L.A., Huey, B., and King, M.-C. (1990). Linkage of early-onset familial breast cancer to chromosome 17q21. Science *250*, 1684–1689.

Harper, J.W., and Elledge, S.J. (2007). The DNA damage response: ten years after. Mol. Cell *28*, 739–745.

Hernández, H., and Robinson, C.V. (2007). Determining the stoichiometry and interactions of macromolecular assemblies from mass spectrometry. Nat. Protoc. *2*, 715–726.

Hu, X., Kim, J.A., Castillo, A., Huang, M., Liu, J., and Wang, B. (2011). NBA1/MERIT40 and BRE interaction is required for the integrity of two distinct deubiquitinating enzyme BRCC36-containing complexes. J. Biol. Chem. *286*, 11734–11745.

Hu, X., Paul, A., and Wang, B. (2012). RAP80 recruitment to DNA double strand breaks requires binding to both sumo- and ubiquitin-conjugates. J. Biol. Chem. *287*, 25510–25519.

Huen, M.S.Y., and Chen, J. (2008). The DNA damage response pathways: at the crossroad of protein modifications. Cell Res. *18*, 8–16.

Kabsch, W. (2010). XDS. Acta Crystallogr. D Biol. Crystallogr. *66*, 125–132.

Kim, H., Huang, J., and Chen, J. (2007a). CCDC98 is a BRCA1-BRCT domain-binding protein involved in the DNA damage response. Nat. Struct. Mol. Biol. *14*, 710–715.

Kim, H., Chen, J., and Yu, X. (2007b). Ubiquitin-binding protein RAP80 mediates BRCA1-dependent DNA damage response. Science *316*, 1202–1205.

Kolas, N.K., Chapman, J.R., Nakada, S., Ylanko, J., Chahwan, R., Sweeney, F.D., Panier, S., Mendez, M., Wildenhain, J., Thomson, T.M., et al. (2007). Orchestration of the DNA-damage response by the RNF8 ubiquitin ligase. Science *318*, 1637–1640.

Lee, J.-H.H., and Paull, T.T. (2004). Direct activation of the ATM protein kinase by the Mre11/Rad50/Nbs1 complex. Science *304*, 93–96.

Leung, C.C.Y., and Glover, J.N.M. (2011). BRCT domains: easy as one, two, three. Cell Cycle *10*, 2461–2470.

Liu, X., and Ladias, J.A.A. (2013). Structural basis for the BRCA1 BRCT interaction with the proteins ATRIP and BAAT1. Biochemistry *52*, 7618–7627.

Liu, Z., Wu, J., and Yu, X. (2007). CCDC98 targets BRCA1 to DNA damage sites. Nat. Struct. Mol. Biol. *14*, 716–720.

Mailand, N., Bekker-Jensen, S., Faustrup, H., Melander, F., Bartek, J., Lukas, C., and Lukas, J. (2007). RNF8 ubiquitylates histones at DNA double-strand breaks and promotes assembly of repair proteins. Cell *131*, 887–900.

Manke, I.A., Lowery, D.M., Nguyen, A., and Yaffe, M.B. (2003). BRCT repeats as phosphopeptide-binding modules involved in protein targeting. Science *302*, 636–639.

Miki, Y., Swensen, J., Shattuck-Eidens, D., Futreal, P.A., Harshman, K., Tavtigian, S., Liu, Q., Cochran, C., Bennett, L.M., Ding, W., et al. (1994).

A strong candidate for the breast and ovarian cancer susceptibility gene BRCA1. Science *266*, 66–71.

Rodriguez, M., Yu, X., Chen, J., and Songyang, Z. (2003). Phosphopeptide binding specificities of BRCA1 COOH-terminal (BRCT) domains. J. Biol. Chem. *278*, 52914–52918.

Sato, Y., Yoshikawa, A., Mimura, H., Yamashita, M., Yamagata, A., and Fukai, S. (2009). Structural basis for specific recognition of Lys 63-linked polyubiquitin chains by tandem UIMs of RAP80. EMBO J. *28*, 2461–2468.

Shakya, R., Reid, L.J., Reczek, C.R., Cole, F., Egli, D., Lin, C.-S., DeRooij, D.G., Hirsch, S., Ravi, K., Hicks, J.B., et al. (2011). BRCA1 tumor suppression depends on BRCT phosphoprotein binding, but not its E3 ligase activity. Science *334*, 525–528.

Shattuck-Eidens, D., McClure, M., Simard, J., Labrie, F., Narod, S., Couch, F., Hoskins, K., Weber, B., Castilla, L., Erdos, M., et al. (1995). A collaborative survey of 80 mutations in the BRCA1 breast and ovarian cancer susceptibility gene. Implications for presymptomatic testing and screening. JAMA *273*, 535–541.

Shen, Y., and Tong, L. (2008). Structural evidence for direct interactions between the BRCT domains of human BRCA1 and a phospho-peptide from human ACC1. Biochemistry *47*, 5767–5773.

Shiozaki, E.N., Gu, L., Yan, N., and Shi, Y. (2004). Structure of the BRCT repeats of BRCA1 bound to a BACH1 phosphopeptide: implications for signaling. Mol. Cell *14*, 405–412.

Sobott, F., Hernández, H., McCammon, M.G., Tito, M.A., and Robinson, C.V. (2002). A tandem mass spectrometer for improved transmission and analysis of large macromolecular assemblies. Anal. Chem. *74*, 1402–1407.

Szabo, C., Masiello, A., Ryan, J.F., and Brody, L.C. (2000). The breast cancer information core: database design, structure, and scope. Hum. Mutat. *16*, 123–131.

Uziel, T., Lerenthal, Y., Moyal, L., Andegeko, Y., Mittelman, L., and Shiloh, Y. (2003). Requirement of the MRN complex for ATM activation by DNA damage. EMBO J. *22*, 5612–5621.

Varma, A.K., Brown, R.S., Birrane, G., and Ladias, J.A.A. (2005). Structural basis for cell cycle checkpoint control by the BRCA1-CtIP complex. Biochemistry *44*, 10941–10946.

Wang, B. (2012). BRCA1 tumor suppressor network: focusing on its tail. Cell Biosci. *2*, 6.

Wang, B., and Elledge, S.J. (2007). Ubc13/Rnf8 ubiquitin ligases control foci formation of the Rap80/Abraxas/Brca1/Brcc36 complex in response to DNA damage. Proc. Natl. Acad. Sci. USA *104*, 20759–20763.

Wang, B., Matsuoka, S., Ballif, B.A., Zhang, D., Smogorzewska, A., Gygi, S.P., and Elledge, S.J. (2007). Abraxas and RAP80 form a BRCA1 protein complex required for the DNA damage response. Science *316*, 1194–1198.

Wang, B., Hurov, K., Hofmann, K., and Elledge, S.J. (2009). NBA1, a new player in the Brca1 A complex, is required for DNA damage resistance and checkpoint control. Genes Dev. *23*, 729–739.

Williams, R.S., and Glover, J.N.M. (2003). Structural consequences of a cancer-causing BRCA1-BRCT missense mutation. J. Biol. Chem. *278*, 2630–2635.

Williams, R.S., Green, R., and Glover, J.N.M. (2001). Crystal structure of the BRCT repeat region from the breast cancer-associated protein BRCA1. Nat. Struct. Biol. *8*, 838–842.

Williams, R.S., Lee, M.S., Hau, D.D., and Glover, J.N.M. (2004). Structural basis of phosphopeptide recognition by the BRCT domain of BRCA1. Nat. Struct. Mol. Biol. *11*, 519–525.

Winn, M.D., Ballard, C.C., Cowtan, K.D., Dodson, E.J., Emsley, P., Evans, P.R., Keegan, R.M., Krissinel, E.B., Leslie, A.G.W., McCoy, A., et al. (2011). Overview of the CCP4 suite and current developments. Acta Crystallogr. D Biol. Crystallogr. *67*, 235–242.

Wong, A.K., Ormonde, P.A., Pero, R., Chen, Y., Lian, L., Salada, G., Berry, S., Lawrence, Q., Dayananth, P., Ha, P., et al. (1998). Characterization of a carboxy-terminal BRCA1 interacting protein. Oncogene *17*, 2279–2285.

Wu, J., Huen, M.S., Lu, L.-Y.Y., Ye, L., Dou, Y., Ljungman, M., Chen, J., and Yu, X. (2009). Histone ubiquitination associates with BRCA1-dependent DNA damage response. Mol. Cell. Biol. *29*, 849–860.

Wu, Q., Jubb, H., and Blundell, T.L. (2015). Phosphopeptide interactions with BRCA1 BRCT domains: More than just a motif. Prog. Biophys. Mol. Biol. *117*, 143–148.

Yu, X., Wu, L.C., Bowcock, A.M., Aronheim, A., and Baer, R. (1998). The C-terminal (BRCT) domains of BRCA1 interact in vivo with CtIP, a protein implicated in the CtBP pathway of transcriptional repression. J. Biol. Chem. *273*, 25388–25392.

Yu, X., Chini, C.C.S., He, M., Mer, G., and Chen, J. (2003). The BRCT domain is a phospho-protein binding domain. Science *302*, 639–642.

Molecular Cell

Supplemental Information

Structure of BRCA1-BRCT/Abraxas Complex Reveals Phosphorylation-Dependent BRCT Dimerization at DNA Damage Sites

Qian Wu, Atanu Paul, Dan Su, Shahid Mehmood, Tzeh Keong Foo, Takashi Ochi, Emma L. Bunting, Bing Xia, Carol V. Robinson, Bin Wang, and Tom L. Blundell

Supplemental Information

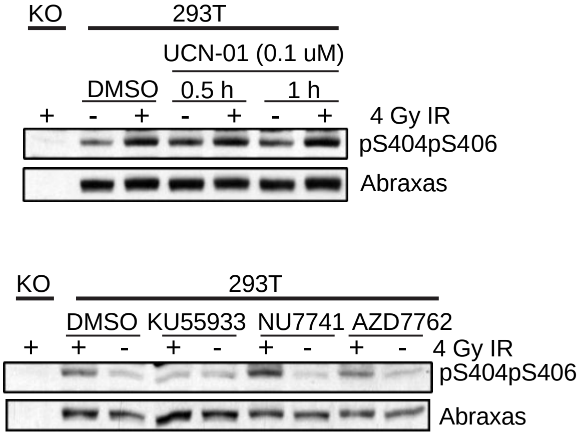

Figure S1 (related to Figure 1): IR-induced double phosphorylation of Abraxas C-terminus S404 and S406 is ATM-dependent, not ATR-, DNAPK-, Chk1- or Chk2 -dependent. Cells were incubated with Chk1 inhibitor (UCN-01), Chk1/2 inhibitor (AZD7762, 10 uM), ATM inhibitor (KU55933, 10 uM), DNA-PK inhibitor (NU7741, 10uM) for 2 hr at indicated concentrations. Cells were then either exposed to 4 Gy IR or untreated followed by 1 hr incubation at 37°C. Abraxas pS404pS406 levels were determined by Western blot.

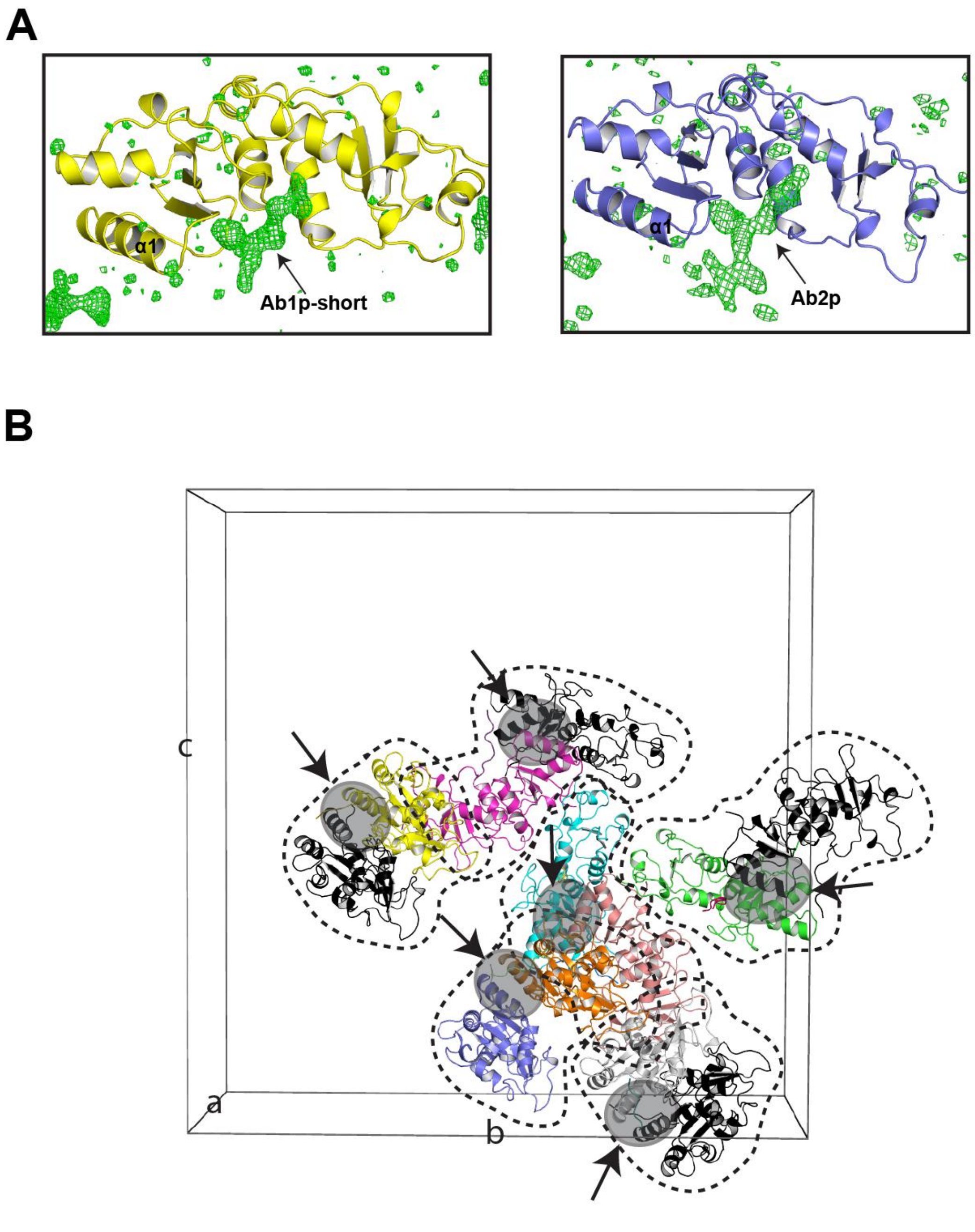

Figure S2 (related to Figures 2 and 3): Electron density for Abraxas phosphopeptide and arrangement of BRCT-Ab2p molecules in the crystal asymmetric unit. A) *Fo-Fc* map (σ=3.0) is shown for Ab1p_short and Ab2p. B) 8 copies of BRCT-Ab2p in one asymmetric unit of the cell (space group $P2_12_12_1$) were coloured in yellow, magenta, cyan, tint, green, orange, blue and grey individually. The same dimer interface occurs in each dimer of the BRCT-Ab2p complex. BRCT-Ab2p molecules belong to different ASUs are coloured in black. Each BRCT-Ab2p complex dimer unit was circulated in dashed line. The dimer interface is highlighted in grey and indicated by a black arrow.

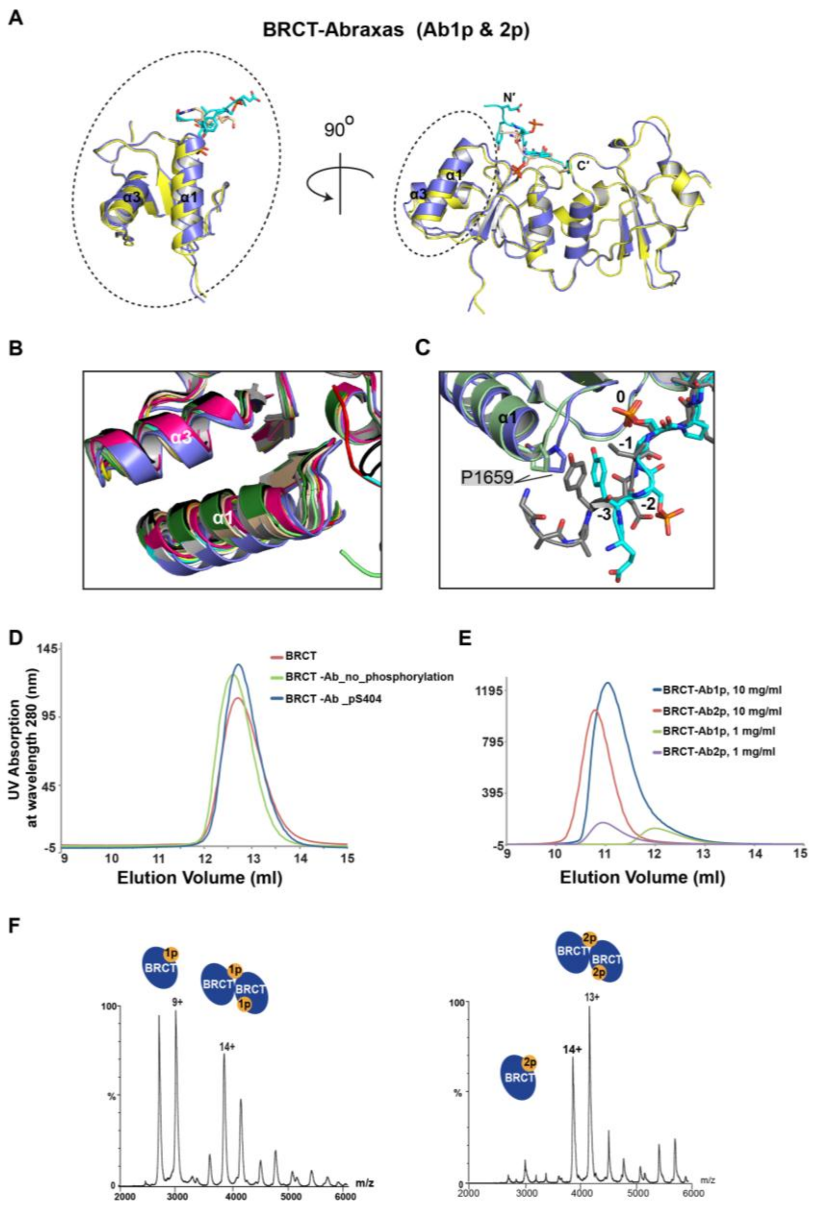

Figure S3 (related to Figure 3): Comparison of BRCT-Abraxas structures with other related BRCA1-BRCT structures, further experiments on the BRCA1-Abraxas complex in solution and native mass spectrometry. A) Superimposition of both BRCT-Ab1p_short (BRCT in yellow and Ab1p_short in wheat colour) and BRCT–Ab2p (BRCT in blue and Ab2p in cyan) structures by aligning the pSPTF motif and the BRCT binding pocket (α2, α1′ and α3′). N- and C-termini are indicated. The black dashed circle highlights the relative structural movements in α1 and α3 and is further zoomed in and rotated 90 degrees. B) Superimposition of BRCT-Ab2p with other BRCT related structures: BRCT-Ab2p (blue), BRCT-Ab1p_short (yellow), BRCT-Bach1 (PDB: 1T15 (cyan) and 1T29 (red) (Clapperton et al., 2004; Shiozaki et al., 2004), BRCT with tetrapeptide motifs (PDB: 3K0K (orange) and 3K0H (wheat)) (Campbell et al., 2010), BRCT with acetyl-coA carboxylase 1 peptide (PDB:3COJ (pink)) (Shen and Tong, 2008) , BRCT-CtIP (PDB: 1Y98 (black)) (Varma et al., 2005), ATRIP (PDB: 4IGK (dark green)) (Liu and Ladias, 2013), optimized peptide (PDB:1T2V (light green)) (Williams et al., 2004) and BAAT1 (PDB:4IFI (white)) (Liu and Ladias, 2013). C) Superimposition of BRCT-Ab2p with BRCT-Optimized peptide (PDB code: 1T2V). The positions of related residues are relative to the phosphorylated serine in pSxxF is in the 0 position. D) Elution profile of BRCT only, BRCT-Ab_no_phosphorylation and BRCT-Ab pS404 are superimposed. BRCT, BRCT-Ab_pS404 and BRCT-Ab_no_phosphorylation are eluted at similar positions. E) BRCT-Ab1p and BRCT-Ab2p at high concentration (10 mg/ml). **(**F) Native mass spectra of BRCT-Abraxas complexes. Protein samples are tested at 75 μM concentration.

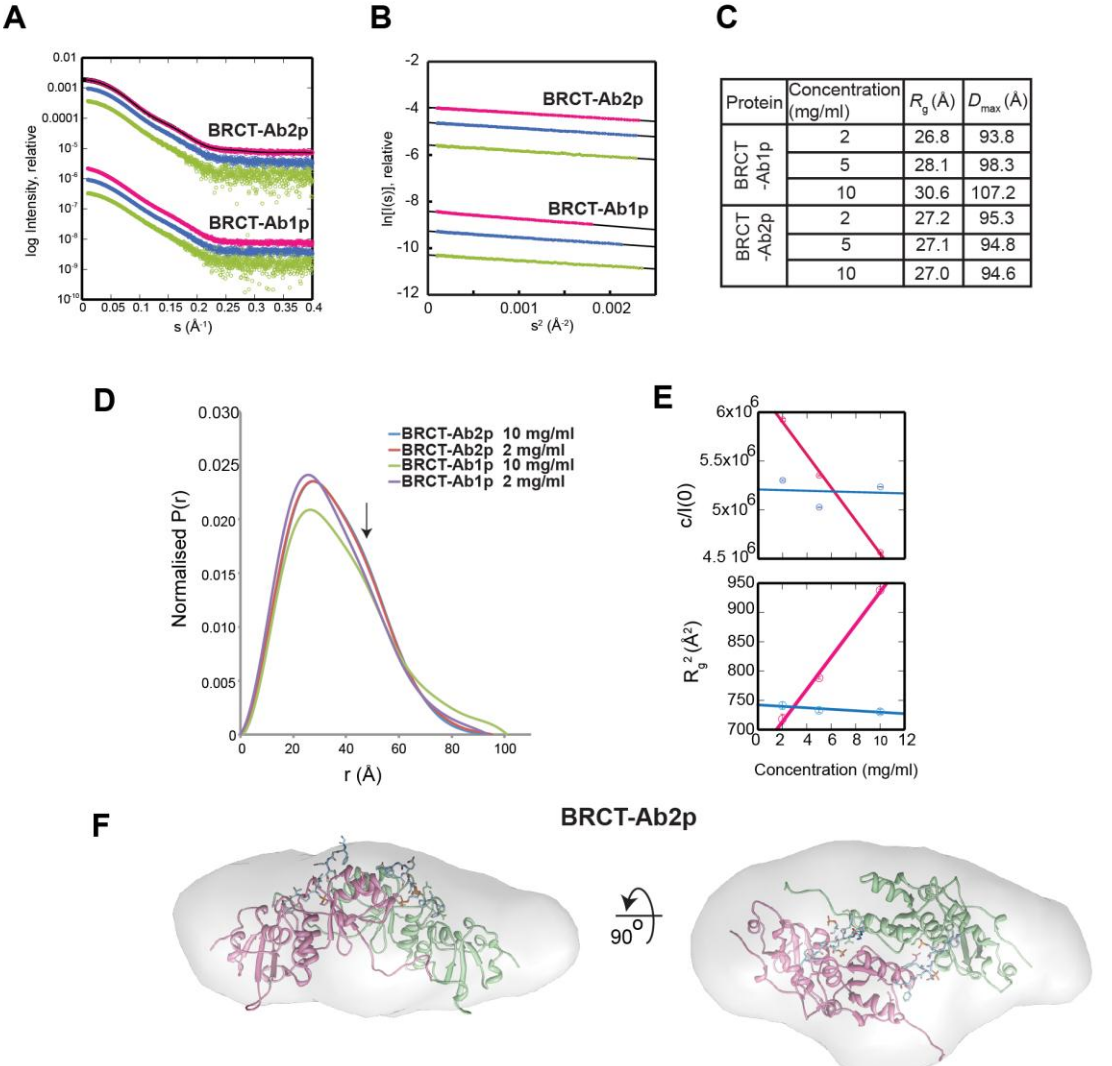

Protein	Concentration (mg/ml)	R_g (Å)	D_{max} (Å)
BRCT -Ab1p	2	26.8	93.8
	5	28.1	98.3
	10	30.6	107.2
BRCT -Ab2p	2	27.2	95.3
	5	27.1	94.8
	10	27.0	94.6

Figure S4 (related to Figure 3). Identification of BRCT-Abraxas using SAXS. A) The SAXS scattering profiles of BRCT-Ab2p at 2 mg/ml (green), 5 mg/ml (blue) and 10 mg/ml (pink) are shifted up compared to those of BRCT-Ab1p for clarity. The black line is simulated-scattering profiles of the crystal structure of BRCT-Ab2p 2:2 dimer complex. B) Guinier plots of BRCT-Ab1p and BRCT-Ab2p. The scattering data (dot; $s<1.3/R_g$) are shown together with linear fit lines (black). C) R_g and D_{max} values of BRCT-Ab1p and BRCT-Ab2p. D) Pairwise distance-distribution functions of BRCT domains. The distribution functions of BRCT-Ab1p at 2 mg/ml (green) and 10 mg/ml (purple) are shown together with that of BRCT-Ab2p 2 mg/ml (red) and BRCT-Ab2p 10 mg/ml (blue). Each distribution function was normalized so that its total area value is 1. E) Concentration dependence of $I(0)$ and radius of gyration R_g of BRCT-Ab1p (pink) and BRCT-Ab2p (blue). The top panel shows the plots of the concentration c versus $c/I(0)$. The lower panel shows the plots of c versus the square of R_g. The top and lower panels share the same abscissa axis. BRCT-Ab1p and BRCT-Ab2p are represented by pink and blue respectively. F) Shape reconstruction of BRCT-Ab2p. The structure of BRCT-Ab2p dimer, of which N-termini were modeled using RapperTK, were fitted in the averaged DAMMIN envelope.

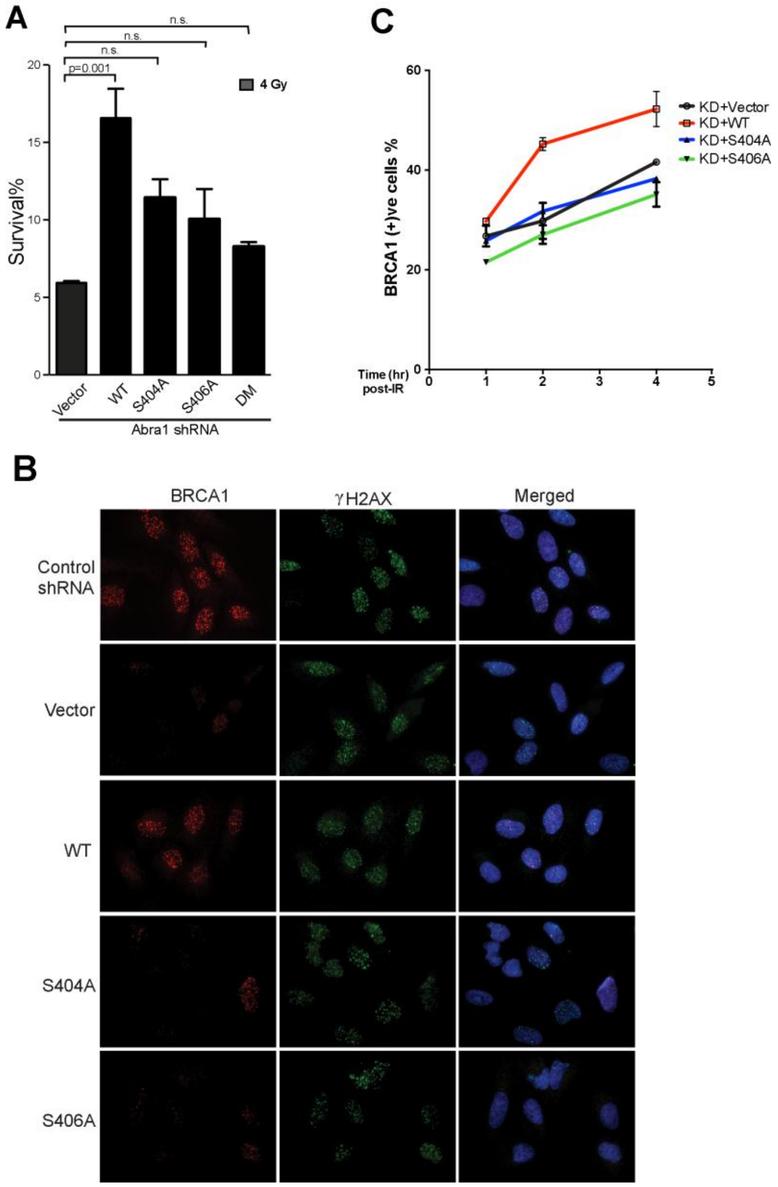

Figure S5 (related to Figure 5): Phosphorylation of S404 and S406 are both important for cellular resistance to IR and BRCA1 accumulation at DNA damage sites. A) An independent colony survival assay for Abraxas-deficient cells expressing WT or mutants of Abraxas treated with 4 Gy IR. Data are presented as means ± s.d. Data analyses are processed by ANOVA and statistical significance was determined by Tukey’s multiple comparisons test. B) Representative images of BRCA1 IRIF in Abra1 shRNA knockdown cells complemented with vector, wild-type or mutants of Abraxas in response to 10 Gy IR followed by 2 hr incubation at 37°C. C) Quantification of the percentage of cells containing more than 10 BRCA1 IRIF foci at indicated time points after 10 Gy IR followed by incubation at 37°C. More than 300 cells were counted for each experiment. Data are presented as means ± s.d.

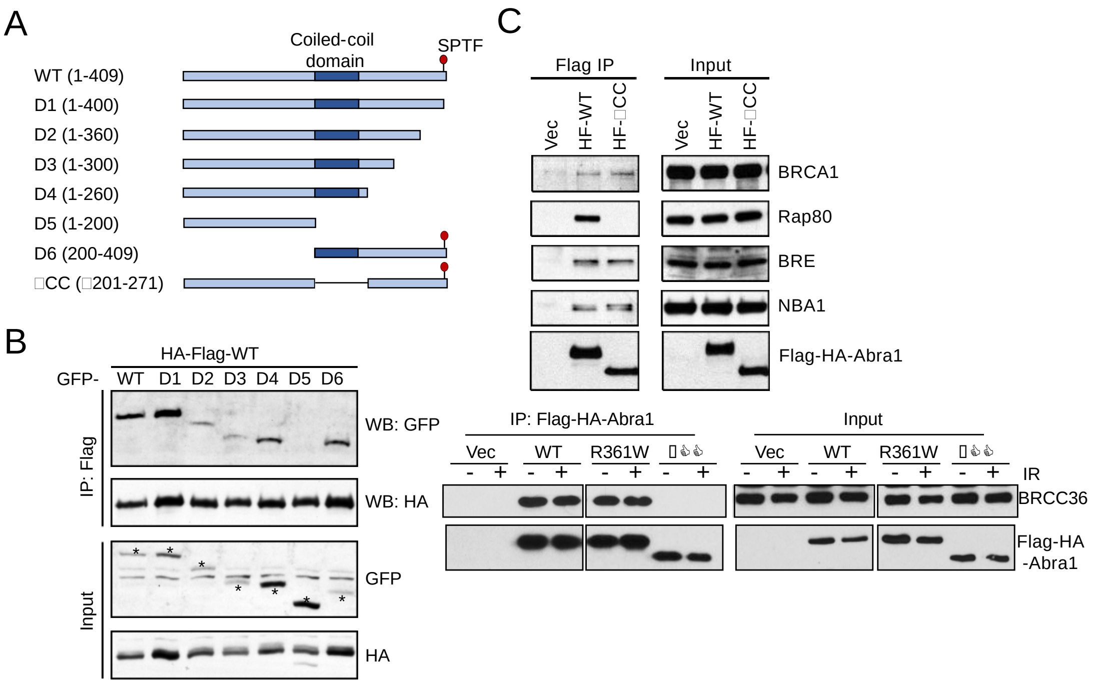

Figure S6 (related to Figure 6): Abraxas forms dimers through the coiled-coil domain independent of binding to BRCA1. A) A schematic view of the Abraxas deletion mutants generated. B) The coiled-coil domain is required for Abraxas dimerization. HA-Flag-tagged wild type (WT) Abraxas were co-transfected with GFP-tagged WT or deletion mutants of Abraxas into 293T cells. 48 h after transfection, cell lysates were prepared and used for Flag-immunoprecipitation. Western blots with GFP or HA antibodies are shown. Expression of GFP-tagged WT or mutant-Abraxas protein products are indicated with "*". C) Abraxas coiled-coil deletion mutant (**Δ**CC) retains interaction with other components of the BRCA1-A complex. HA-Flag-tagged WT or **Δ**CC were transiently expressed in 293T cells. The samples in the upper panel were prepared from cells not treated with ionizing radiation. The lower panel shows the interaction of Abraxas with BRCC36. An Abraxas mutant (R361W) that retains the interaction with BRCC36 was also used as a control. Samples of cells treated with 10 Gy IR or untreated were used to show the interaction of Abraxas with BRCC36. Flag-immunoprecipitation was carried out followed by Western blots with antibodies against indicated protein.

Supplemental Experimental Procedures

Cell Lines, Cell Culture and Antibodies

To generate Abraxas knockdown cells, U2OS cells were infected with retrovirus containing shRNAs against Abraxas followed by selection with puromycin (0.8 ug/ml) for 5 days. The Abraxas knockdown stable cell line was then complemented with expression of empty MSCV vector or expression constructs containing HA-tagged WT or mutant Abraxas, and selected with Blasticidin (9 ug/ml) 1 week for stable expression. 293T Abraxas knockout cells were generated by CRISPR-Cas9 system (Fu et al., 2014; Ran et al., 2013) In brief, 293T cells were infected with lentivirus carrying pLentiCRISPR including Cas9 and sgRNA, which targets Abraxas genomic sequence 5'-GGCGGCGGTAGCATGGA-3'. Cells were then subjected to puromycin selection (2 ug/ml) for three days and plated in low density for culturing for ten days. Single colonies were selected and knockout cells were confirmed by Western blotting with Abraxas antibody and PCR-sequence of the Abraxas genomic locus. Rabbit anti-Abraxas double-phosphorylated pS404pS406 antibody was generated using conjugated GFGEYpSRpSPTF phosphopeptide. The rabbit anti-Abraxas and anti-Abraxas S406 antibodies were generated as previously described (Wang et al., 2007). Other antibodies used were BRCA1 antibodies (D9, Santa Cruz Biotechnologies); GFP antibodies (Invitrogen); γ-tubulin antibodies (Sigma); rabbit anti-HA antibodies (Cell Signaling), Rabbit γH2AX (Upstate), Orc2 (PharMingen).

Immunofluorescence

Abra1 shRNA knockdown cells complemented with empty vector, wild type or mutants of Abra1 were analyzed for BRCA1 IR-induced foci formation (IRIF). At least 500 cells were counted for each cell type and cells containing more than 10 foci were counted as positive. Mean and standard deviations are shown in the Figures 5B,D,E, S5A and statistical analysis was performed by student's t-test or ANOVA with Tukey's multiple comparisons test. p-value is as indicated. All images were obtained with a Nikon TE2000 inverted microscope with a Photometrics Cool- Snap HQ camera. Quantification of BRCA1 was performed using Imaris software (Bitplane). The DAPI channel was used to select the nuclei of the cells in the field, red and green channel were used for BRCA1 and γH2AX, respectively. For BRCA1 foci intensity measurement, foci were defined as particles bigger than 0.25 μM in diameter with an intensity cut-off value (1200) to eliminate background. At least 50 cells were counted and plotted using GraphPad Prism software.

Cell Lysis and Immunoprecipitation

Cells were lysed in NETN buffer (50 mM Tris-HCl, pH 8.0, 150 mM NaCl, 1 mM EDTA, 0.5% Nonidet P-40) with protease inhibitors and protein phosphatase inhibitors, 1 mM NaF, and 1 mM Na_3VO_4. For Flag IP, cell lysates were incubated with Flag beads (Sigma) overnight with gentle agitation at 4°C. The beads were washed with NETN lysis buffer four times and eluted with 3X sample buffer for Western blot analysis. For analyzing Abraxas dimerization *in vivo*, GFP-tagged and HF-tagged Abraxas wildtype, S404A, S406A mutant or coiled-coil deletion mutant were transiently transfected to 293T cells. Two days after transfection, cells were either untreated or exposed to 10 Gy IR. 2 hr later, cells were collected for GFP- or Flag- IP and Western blot was probed with either antibodies against HA or GFP. For analyzing BRCA1 dimerization *in vivo*, Flag- or Myc-tagged BRCA1 full-length wild type or mutants, or HA- and Myc-tagged BRCA1 BRCT fragments were analyzed in a similar way. ATM inhibitor (KU55933), ATR inhibitor (VE-821), DNA-PK inhibitor (NU7441) and Chk1/2 inhibitor (AZD7762) were purchased from Selleckchem and dissolved in dimethyl sulphoxide (DMSO).

Clonogenic Survival Assay

Abra1 shRNA knockdown cells complemented with empty vector, wild type or mutants of Abra1 were analyzed for cell survival in response to IR. Stable U2OS cell lines were seeded at low density in 10 cm dishes and irradiated with 4 Gy ionizing irradiation using a ^{137}Cs source. The cells were then cultured at 37°C for 14 days to allow colonies to form. Colonies were stained with 2% methylene blue and 50% ethanol for 10 min. Colonies containing 50 or more cells were counted as positive and statistical data were analyzed by analysis of variance (ANOVA) with Tukey's multiple comparisons test.

Chromatin fractionation

Cells were irradiated at 10 Gy followed by 1 hr incubation at 37°C. For total cell extracts, cells were lysed in NETN150 buffer containing protease inhibitor mixture and analyzed by Western blot. For

chromatin fractionation, irradiated cells were washed in PBS and resuspended in Buffer A (10 mM Hepes pH 7.9, 10 mM KCl, 1.5 mM $MgCl_2$, 0.34 M sucrose, 10% glycerol, 5 mM NaF, 1 mM Na_3VO_4 , 1 mM dithiothreitol (DTT), and protease inhibitor mixture) containing 0.1% Triton X-100, and incubated on ice for 5 min for permeabilization. The cytosolic fraction was then separated by centrifugation at 4000 rpm for 5 min at 4°C. The supernatant was discarded and the nuclei pellet was washed once with Buffer A and resuspended in Buffer B (3 mM EDTA, 0.2 mM EGTA, 1 mM DTT, protease inhibitor mixture) and incubated for 30 min on ice. The soluble nuclear fraction was separated by centrifugation at 4500 rpm for 5 min. The chromatin fraction pellet was washed with Buffer B and resuspended in 100 μl Laemmli sample buffer and sonicated for 10 sec before analysis.

BRCT constructs, expression and purification

The BRCT domains (residues 1646-1859) of the *BRCA1* gene were amplified from IMAGE cDNA (ID 7961446) using PCR and cloned into the pHAT2 vector (with noncleavable N-terminal His-tag) (Peränen et al., 1996) and transformed into Rosetta^{TM}2 (DE3) cells (Invitrogen). Cell culture was grown in LB medium till the OD_{600} was approximately 0.6. IPTG was added to a final concentration of 1 mM and cells were grown overnight at 16°C. 1 g of harvested cells was resuspended with 10 ml of lysis buffer (50 mM Tris pH 8.0, 300 mM NaCl, 20 mM imidazole and one protease inhibitor tablet per 50 ml lysis buffer). Cells were lysed using sonication. The supernatant after centrifugation was then filtered by 0.45 μm filter and loaded onto HisTrap HP 5ml (GE healthcare) pre-equilibrated with binding buffer (50 mM Tris pH 8.0, 300 mM NaCl and 20 mM imidazole) After washing with 10x column volume of binding buffer and binding buffer with 50 mM imidazole, the protein was eluted using elution buffer (50 mM Tris pH 8.0, 300 mM NaCl and 200 mM imidazole). The eluted BRCT domains were about 80% pure as assessed by 12% SDS-PAGE. Remaining contaminants were removed by gel filtration using a Superdex 75 10/300 equilibrated with Buffer A (20 mM Tris pH 8.0, 150 mM NaCl and 5 mM DTT). Fractions containing pure BRCT sample were analyzed in 12% SDS-PAGE gel, pooled, concentrated and stored at -80°C. All mutants were made by site-directed mutagenesis using wt BRCT in pHAT2 as PCR template. Mutant proteins were expressed and purified using the procedure described above.

Phosphopeptides

All phosphopeptides were synthesized to above 95% purity without modification at both N and C terminus (Biomatik). Peptides were dissolved in Buffer A to 25 mg/ml. The pH of the peptide solution was adjusted to near pH 8 using 0.5 M NaOH. The peptide names and sequences are listed below:
Abraxas peptides: 1)Ab1p: GFGEYSRpSPTF; 2)Ab2p: GFGEYpSRpSPTF; 3)Ab1p_short: YSRpSPTF; 4)Ab2p_short: YpSRpSPTF; 5)Ab_pS404: GFGEYpSRSPTF; 6)Ab_no_phosphorylation: GFGEYSRSPTF; 7)Ab2p(Y403A): GFGEApSRpSPTF; 8)Ab2p(Y403A)_short: ApSRpSPTF; 9)Ab2p(F400D): GDGEYpSRpSPTF; 10)Ab2p(E402R): GFGRYpSRpSPTF; 11)Ab1p(S404P): GFGEYPRpSPTF; 12)Ab1p(S404D): GFGEYDRpSPTF; 13)Bach1: ISRSTpSPTFNKQ; 14) CtIP: PTRVSpSPVFGAT

Protein crystallization and data collection

Crystals were cryoprotected in crystallization solution containing 30% glycerol using the two-step transferring method and then subsequently flash frozen in liquid nitrogen. The structure of BRCT-Bach1 (PDB code 1T15) (Clapperton et al., 2004) without Bach1 phosphopeptide was used as the search model. Relaxed NCS restraints among all the molecules of the BRCT-Ab2p in the asymmetric unit were used in the refinement protocol as defined in phaser_refine module. This resulted in clear $2F_o$-F_c and F_o-F_c electron density that allowed manual building of the Abraxas peptide using *Coot* (Emsley et al., 2010) (Figure S1). For BRCT-Ab2p, the structure connectivity and disordered region vary among molecules in the ASU depending on its position in the crystallographic packing, therefore chains IDs G, H, O and P have poorer electron density map than other chains. Further structure refinements also included simulated annealing, optimizing X-ray/Stereochemistry weight, optimizing X-ray/ADP weight and side chain adjustment using *Coot*. The buried surface area after dimerization of BRCT-Ab2p was calculated using PISA (Krissinel and Henrick, 2007).

Identification of BRCT-Ab1p and BRCT-Ab2p using SAXS

We carried out SAXS of the BRCT-Ab1p and BRCT-Ab2p in different concentrations. The scattering profiles (Figure S4A) of BRCT-Ab1p and BRCT-Ab2p at high concentration (10 mg/ml) were similar and indicated that the samples were monodisperse (Figure S4B). The scattering profiles of BRCT-Ab2p did not change by increasing the concentration of the sample whereas BRCT-Ab1p changed particularly at low angle. We then used the BRCT-Ab2p dimer crystal structure to calculate its

scattering profile and compare it with the observed profile of BRCT-Ab2p (black line in Figure S4A). The calculated-scattering profiles of the 2:2 BRCT-Ab2p dimer explain the observed scattering profiles much better than for the 1:1 complex (χ=1.675 and 3.806 respectively) indicating that BRCT-Ab2p is dimerized in solution. The fitting of the 2:2 dimer structure against the scattering profile of BRCT-Ab1p was better at 10 mg/ml (χ=1.934) than at 2 mg/ml (χ=5.975). The pairwise distance-distribution functions of BRCT-Abraxas complexes showed that BRCT-Ab2p has a very similar profile at both 10 mg/ml and 2 mg/ml concentrations. They both have a shoulder around 50 Å (indicated by black arrow in Figure S4D), which is clearer than that of BRCT-Ab1p at 10 mg/ml and 2 mg/ml. Furthermore, BRCT-Ab1p showed inter-particle attraction by increasing the concentration of the sample but BRCT-Ab2p did not (Figure S4E) (Nakasako et al., 2005). BRCT-Ab2p complex dimer crystal structure can be fitted into the envelope of an averaged DAMMIN model of the dimer (Figure S4F).

SAXS analysis
SAXS data of 2, 5 and 10 mg/ml BRCT-Ab1p and BRCT-Ab2p in Buffer A (20 mM Tris pH 8.0, 150 mM NaCl and 5 mM DTT) were collected at I22 in Diamond Light Source. Protein samples were loaded into glass capillaries using a BIOSAXS robot and the scattering intensities of the sample were measured at 5°C using the Pilatus 2M detector. The scattering vector s is $4\pi\sin\theta/\lambda$, where θ is half of the scattering angle and λ is the wavelength (0.9987 Å). Data reduction was carried out using DAWN. Primus was used to interpolate to the zero concentration of BRCT-Abraxas2p data using the ZeroConc module and to calculate R_g & $I(0)$ using the AutoRg module and pair-distribution functions & D_{max} using the AutoGNOM module (Figure S4C) (Petoukhov et al., 2012). Ten dummy-residue models of BRCT-Ab2p were created using DAMMIN (Svergun, 1999) and were superimposed and averaged using SUPCOMB (Kozin and Svergun, 2001) and DAMAVER (Volkov and Svergun, 2003) to generate a dummy-residue model of BRCT-Ab2p. The mean value of normalized-spatial discrepancy was 0.567. The selection of the best-fit model was conducted as follows. The missing N-termini of the BRCT domains and the peptide from BRCT-Ab2p crystal structure were added by *Coot* (Emsley et al., 2010). RapperTK (Gore et al., 2007) was used to generated 100 conformations of these flexible structures. A conformation of each chain was randomly selected and mixed to generate 1000 conformations of BRCT-Ab2p. The fitting of each conformation was calculated using CRYSOL to select the best conformation.

Supplemental References

Campbell, S.J., Edwards, R.A., and Glover, J.N.M. (2010). Comparison of the structures and peptide binding specificities of the BRCT domains of MDC1 and BRCA1. Structure *18*, 167–176.

Clapperton, J.A., Manke, I.A., Lowery, D.M., Ho, T., Haire, L.F., Yaffe, M.B., and Smerdon, S.J. (2004). Structure and mechanism of BRCA1 BRCT domain recognition of phosphorylated BACH1 with implications for cancer. Nat. Struct. Mol. Biol. *11*, 512–518.

Emsley, P., Lohkamp, B., Scott, W.G., and Cowtan, K. (2010). Features and development of Coot. Acta Crystallogr. D. Biol. Crystallogr. *66*, 486–501.

Fu, Y., Sander, J.D., Reyon, D., Cascio, V.M., and Joung, J.K. (2014). Improving CRISPR-Cas nuclease specificity using truncated guide RNAs. Nat Biotech *32*, 279–284.

Gore, S.P., Karmali, A.M., and Blundell, T.L. (2007). Rappertk: a versatile engine for discrete restraint-based conformational sampling of macromolecules. BMC Struct. Biol. *7*.

Kozin, M.B., and Svergun, D.I. (2001). Automated matching of high- and low-resolution structural models. J. Appl. Crystallogr. *34*, 33–41.

Krissinel, E., and Henrick, K. (2007). Inference of macromolecular assemblies from crystalline state. J. Mol. Biol. *372*, 774–797.

Liu, X., and Ladias, J.A.A. (2013). Structural basis for the BRCA1 BRCT interaction with the proteins ATRIP and BAAT1. Biochemistry *52*, 7618–7627.

Nakasako, M., Iwata, T., Inoue, K., and Tokutomi, S. (2005). Light-induced global structural changes in phytochrome A regulating photomorphogenesis in plants. FEBS J. *272*, 603–612.

Peränen, J., Rikkonen, M., Hyvönen, M., and Kääriäinen, L. (1996). T7 vectors with modified T7lac promoter for expression of proteins in Escherichia coli. Anal. Biochem. *236*, 371–373.

Petoukhov, M. V., Franke, D., Shkumatov, A. V., Tria, G., Kikhney, A.G., Gajda, M., Gorba, C., Mertens, H.D.T., Konarev, P. V., and Svergun, D.I. (2012). New developments in the ATSAS program package for small-angle scattering data analysis. J. Appl. Crystallogr. *45*, 342–350.

Ran, F.A., Hsu, P.D., Wright, J., Agarwala, V., Scott, D.A., and Zhang, F. (2013). Genome engineering using the CRISPR-Cas9 system. Nat. Protoc. *8*, 2281–2308.

Shen, Y., and Tong, L. (2008). Structural evidence for direct interactions between the BRCT domains of human BRCA1 and a phospho-peptide from human ACC1. Biochemistry *47*, 5767–5773.

Shiozaki, E.N., Gu, L., Yan, N., and Shi, Y. (2004). Structure of the BRCT repeats of BRCA1 bound to a BACH1 phosphopeptide: implications for signaling. Mol. Cell *14*, 405–412.

Svergun, D.I. (1999). Restoring Low Resolution Structure of Biological Macromolecules from Solution Scattering Using Simulated Annealing. *76*, 2879–2886.

Varma, A.K., Brown, R.S., Birrane, G., and Ladias, J.A.A. (2005). Structural Basis for Cell Cycle Checkpoint Control by the BRCA1−CtIP Complex. Biochemistry *44*, 10941–10946.

Volkov, V. V, and Svergun, D.I. (2003). Uniqueness of ab initio shape determination in small-angle scattering. J. Appl. Crystallogr. *36*, 860–864.

Wang, B., Matsuoka, S., Ballif, B.A., Zhang, D., Smogorzewska, A., Gygi, S.P., and Elledge, S.J. (2007). Abraxas and RAP80 Form a BRCA1 Protein Complex Required for the DNA Damage Response. Science (80-.). *316*, 1194–1198.

Williams, R.S., Lee, M.S., Hau, D.D., and Glover, J.N.M. (2004). Structural basis of phosphopeptide recognition by the BRCT domain of BRCA1. Nat Struct Mol Biol *11*, 519–525.
